# Bivalent CD47 Immunotoxin for Targeted Therapy of Lung Cancer

**DOI:** 10.3390/biomedicines14102180

**Published:** 2026-09-26

**Authors:** Jihong Ma, Dinoop Ravindran Menon, Zhaohui Wang, Danielle Mintzlaff, Hugh Rock, Lauren Giesy, Christene A. Huang, David W. Mathes, Trevor Nydam, Elizabeth A. Pomfret, D. Ross Camidge, Hatim E. Sabaawy, Sharon R. Pine, Zhirui Wang

**Affiliations:** 1 Division of Plastic and Reconstructive Surgery, Department of Surgery, School of Medicine, University of Colorado Anschutz Medical Campus, Aurora, CO 80045, USA; 2 Division of Transplant Surgery, Department of Surgery, School of Medicine, University of Colorado Anschutz Medical Campus, Aurora, CO 80045, USA; 3 Division of Medical Oncology, Department of Medicine, School of Medicine, University of Colorado Anschutz Medical Campus, Aurora, CO 80045, USA

**Keywords:** bivalent CD47 immunotoxin, Bi-CD47-IT, CD47, lung cancer, non-small cell lung cancer, NSCLC, targeted therapy, diphtheria toxin

## Abstract

**Background/Objectives**: Lung cancer is the leading cause of cancer-related death in the world, with ~2.5 million people diagnosed and ~1.5 million deaths each year. While the last two decades have yielded substantial progress, with systemic targeted and immune therapies improving treatment responses in subgroups of patients with advanced and refractory lung cancer, there is still a dire unmet clinical need to develop more effective therapies with durable responses. CD47 receptors are overexpressed on the surface of a variety of malignant tumor cells, including lung cancer. Although there has been increasing interest in developing targeting antibodies against CD47 for immunotherapy, they failed clinical trials due to their dose-limiting toxicities, primarily hematopoietic toxicity. **Methods:** Recently, using a unique diphtheria toxin-resistant *Pichia pastoris* yeast expression system, we developed a diphtheria toxin-based bivalent CD47 immunotoxin (bi-CD47-IT) for targeted therapy of CD47^+^ cancers. In this study, we extended application of the targeted therapy bi-CD47-IT to a solid tumor, specifically lung cancer. **Results:** Bi-CD47-IT demonstrated compelling preclinical efficacy in multiple non-small cell lung cancer (NSCLC) cell line-derived xenograft (CDX) and patient-derived xenograft (PDX) mouse models, including subcutaneous, orthotopic, metastatic, and humanized models. **Conclusions**: This study demonstrates the remarkable preclinical activity of bi-CD47-IT against various lung cancer models, making bi-CD47-IT a novel and promising therapeutic approach for NSCLC.

## 1. Introduction

Lung cancer remains the leading cause of cancer-related mortality worldwide, with nearly 2.5 million new cases reported and over 1.5 million deaths annually [1]. The overall 5-year survival rate for advanced non-small cell lung cancer (NSCLC) varies from 9 to 12%. Improvements in systemic therapy, including antibodies, cytotoxic chemotherapy, and especially tyrosine kinase inhibitors (TKIs), have modestly extended overall survival. However, the vast majority of patients treated with systemic targeted therapies will experience progression, and only about 20% of advanced NSCLC patients treated with immunotherapy plus chemotherapy survive up to 5 years [2,3]. Consequently, prognosis for advanced NSCLC remains dismal.

The CD47/SIRPa (CD172a) interaction is one of the most well-characterized immune checkpoints that control macrophages in tumors [4]. CD47 is highly expressed on many cancers, including lung cancer, and it binds to SIRPα, a receptor expressed by macrophages and other myeloid cells. When CD47 and SIRPα engage, inhibitory signals are activated by macrophages to prevent phagocytosis. Expression of CD47 in cancer cells can be controlled by cytokines, oncogenes, and micro-RNAs [5]. Studies have shown that EGFR-mutant tumors are enriched for high CD47 expression at both the mRNA and protein levels [6,7,8]. Recent studies established oncogenic mechanisms of CD47 expression regulation by lung cancer oncogenic drivers, EGFR and KRAS [9,10]. In addition to oncogenes and cytokines, clinical studies demonstrated that standard-of-care lung cancer therapies upregulate CD47 expression, many through the JAK/STAT pathway [11,12]. CD47 in tumor cells demonstrated interactions with proteins that collectively influence cell death and survival, migration, invasion, and angiogenesis [13]. High CD47 expression in NSCLC was shown in large cohorts of patients to be associated with advanced tumor stage and poor patient survival [6,7,14].

The CD47-SIRPα axis is a highly recognized target for developing blocking monoclonal antibodies and recombinant fusion proteins for cancer immunotherapy. Previous approaches were designed to block the interaction between CD47 and SIRPα to relieve suppression of cancer cell phagocytosis by antigen-presenting cells in order to stimulate tumor immunity. However, monotherapy with previous CD47 blockade was ineffective in human clinical trials against many tumor types [15]. Moreover, both CD47 mAbs and SIRPα–Fc fusion proteins have generally been well-tolerated in patients, but grade 3–4 toxicities due to CD47 blockade on RBCs, platelets, and neutrophils occurred in 18% of patients [16], with the most common being thrombocytopenia, neutropenia, and anemia, consistent with CD47 expression on platelets, neutrophils, and RBCs, respectively. Fc-FcγR interactions are required for the antitumor activities of anti-CD47 antibodies [17]. Using a unique diphtheria toxin-resistant *Pichia pastoris* yeast expression system [18], we developed a truncated diphtheria toxin DT390-based bivalent anti-human CD47 immunotoxin (bi-CD47-IT) for targeted therapy of CD47^+^ cancers utilizing the immunotoxin (IT) approach [19]. We genetically engineered bi-CD47-IT to contain two-tandem anti-human CD47 scFv moieties fused to a truncated diphtheria toxin, DT390 [20]. In a prior study, we demonstrated that bi-CD47-IT showed compelling in vivo efficacy against T-cell acute lymphoblastic leukemia (T-ALL) with very low toxicity due to the low binding avidity of bi-CD47-IT to CD47. Bi-CD47-IT significantly prolonged the median survival of T-ALL tumor-bearing mice and highly effectively depleted the T-ALL blast cells in the peripheral blood, spleen, liver, bone marrow, brain, and spinal cord in all tested early and overt T-ALL CDX and PDX mouse models [20]. Because CD47 is also highly expressed in lung cancer cells, in this study, we extended the application of the targeted therapy bi-CD47-IT to a solid tumor, lung cancer.

## 2. Materials and Methods

### 2.1. Human NSCLC Cell Lines, Human NSCLC PDX Samples, Bivalent CD47 Immunotoxin, and Antibodies

Human NSCLC cell lines (A549, RRID: CVCL_0023; H1299, RRID: CVCL_0060; H596, RRID: CVCL_1571; Calu-1, RRID: CVCL_0608; H1975, RRID: CVCL_1511; H1355: RRID: CVCL_1464; H322, RRID: CVCL_1556; H1944, RRID: CVCL_1508; H1573: RRID: CVCL_1478) and normal non-tumorigenic human bronchial epithelial cell lines (BEAS-2B, RRID: CVCL_0168 and HBET2, RRID: CVCL_V079) were obtained from ATCC (Manassas, VA, USA). CUTO41-LR cells were obtained from the University of Colorado Cell Technologies Shared Resource and were grown in the presence of 3 µM of Lorlatinib for one year to maintain resistance to Lorlatinib. Human NSCLC PDX1 (CT257-F4) and PDX3 (220904-M3) were obtained from Apar Pataer (MD Anderson Cancer Center, Houston, TX, USA). PDX3 has an EGFR del exon 19 mutation with MET amplification. The tumor was excised from the patient after progression on osimertinib and tepotinib. Human NSCLC PDX2 (220900-M3) was generated from an EML4-ALK fusion-positive patient who progressed on ALK-targeted therapy at the University of Colorado. Flow cytometry analysis confirmed that the three NSCLC PDX samples were CD47^+^ (Supplementary Figure S6E,F). The NSCLC PDX tumors were expanded in immunodeficient NSG mice. When the NSCLC PDX tumor volume reached the maximum allowed volume, the tumor-bearing mice were euthanized and the PDX tumors were harvested, cut into small pieces, and directly implanted into the dorsal flank of the experimental NSG mice (RRID: IMSR_JAX:005557). Bivalent CD47 immunotoxin (bi-CD47-IT) was produced in our laboratory [20]. The antibodies used in this study, including their RRIDs, are listed in Supplementary Table S1.

### 2.2. Alexa Fluor 488-Labeling of Bi-CD47-IT

Bi-CD47-IT was labeled using Alexa Fluor 488 microscale protein labeling kit (Thermo Fisher Scientific, Catalog: A30006, RRID: AB_2571954, Waltham, MA, USA) following the manufacturer’s instruction. In brief, bi-CD47-IT was reduced to a concentration of ~1 mg/mL. The 1/10 volume of sodium bicarbonate solution was added to the protein solution and mixed well. An appropriate volume of Alexa Fluor 488 reactive dye solution was added according to the equation in the manufacturer’s instructions and incubated for 15 min at room temperature. The conjugate reaction mixture was uploaded onto the resin bed surface and centrifuged at 16,000× *g* for 1 min. The purified dye-labeled bi-CD47-IT was collected into an Eppendorf tube. The labeled immunotoxin concentration was measured using nanodrop (Thermo Fisher Scientific, Waltham, MA, USA) and stored at 4 °C for use.

### 2.3. Flow Cytometry Equilibrium Binding and K_D_ Determination

To evaluate the broad binding profile of bi-CD47-IT, equilibrium binding assays were performed on human red blood cells, human lymphocytes, human monocytes, normal human bronchial epithelial cells, and CD47^+^ NSCLC cells. Cells ($1\times 10^{6}$ cells/sample) were aliquoted into 12 × 75 mm tubes and incubated with serial dilutions of Alexa Fluor 488-labeled bi-CD47-IT, spanning a wide concentration range (1 to 1000 nM) for 30 min at 4 °C. FITC-labeled anti-human CD47 mAb (BD Biosciences, Cat# 556045, San Jose, CA, USA) at 200 nM was used as a positive control, and Alexa Fluor 488-labeled mouse IgG1 isotype at 200 nM served as a negative control. Following incubation, cells were washed twice with 2 mL of cold-flow cytometry buffer and centrifuged at 300× *g* for 5 min. The supernatants were discarded, and cell pellets were resuspended in 300 µL of flow cytometry buffer. To exclude dead cells, 10 µL of 7-AAD was added and incubated at room temperature for 10 min. Flow cytometry was carried out using a CytoFLEX Flow Cytometer (Beckman Coulter, Brea, CA, USA; RRID: SCR_019627), and data were analyzed using FlowJo™ Software (for Windows, Version 10, Becton, Dickinson and Company, Ashland, OR, USA; RRID: SCR_008520).

Equilibrium K_D_ determination was performed based on the median fluorescence intensity (MFI) using nonlinear regression with a saturation binding equation in GraphPad Prism 11 (San Diego, CA; RRID: SCR_002798). The MFI was plotted versus the Alexa Fluor 488-labeled bi-CD47-IT concentrations. Nonlinear regression was calculated using the equation Y = B_max_ × X/(K_D_ + X), where Y is the background-subtracted MFI, X is the bi-CD47-IT concentration, and B_max_ is the maximum specific binding.

### 2.4. Flow Cytometry On-Cell Binding Kinetics Analysis

To deeply investigate the physical and chemical binding mechanisms underlying the cytotoxicity in the primary target tumor lines, rigorous on-cell kinetic assays were performed specifically on human NSCLC cell lines A549 and H1299. Cells ($1\times 10^{6}$ cells/sample) were evaluated using Alexa Fluor 488-labeled bi-CD47-IT at a targeted range of concentrations (100, 200, 500, and 1000 nM), with the aforementioned positive and isotype controls included. To assess time-dependent binding, the cells were incubated with the labeled bi-CD47-IT at 4 °C for intervals of 5, 15, 30, 45, and 60 min. Cells were subsequently washed, stained with 7-AAD, and acquired on the CytoFLEX Flow Cytometer as described above.

For kinetic parameter determination, time-course binding data were organized into an XY data table in GraphPad Prism 11, where X represented the time point (minutes) and Y represented the background-subtracted MFI. The association (K_on_) and dissociation (K_off_) rate constants were determined by nonlinear regression using the ‘association kinetics (two or more concentrations of hot ligand)’ model. The precise on-cell kinetic equilibrium dissociation constant K_D_ for these specific tumor lines was subsequently calculated using the equation K_D_ = K_off_/K_on_.

### 2.5. In Vitro Efficacy Analysis of Bi-CD47-IT

The in vitro efficacy of bi-CD47-IT was determined in human CD47^+^ NSCLC cells using the CellTiter-Glo^®^ Luminescent Cell Viability Assay (Promega, Madison, WI, USA; RRID: SCR_016734) as described previously [21]. This assay measures the luminescence produced by ATP production from metabolically active cells. Increasing concentrations of bi-CD47-IT cause cell death and a corresponding reduction in ATP-related fluorescence. The luminescence signals were recorded using a BioTek Synergy LX Multi-Mode Reader (Agilent, Santa Clara, CA, USA; RRID: SCR_021151). The pCD3-IT (anti-porcine CD3 immunotoxin) was included as negative immunotoxin control. In brief, 1 × 10^4^ tumor cells in a solution of 100 µL were added to each well of the 96-well plate. The serial diluted immunotoxins, starting at 1 × 10^−7^ M to 1 × 10^−14^ M, were added and mixed well to a triple-well set for each diluted immunotoxin. The reaction plate was incubated for 48 h at 37 °C with 5% CO_2_. The plate was equilibrated at room temperature for 30 min. An equal volume of the pre-prepared lyophilized enzyme/substrate CellTiter-Glo mixture was added to each well. The plate was mixed for 2 min at 1000 rpm on a shaker to induce cell lysis. The plate was incubated at room temperature for 10 min to stabilize luminescent signal in the dark. The luminescence signal was measured using the microplate reader (Thermo Fisher Scientific, Waltham, MA, USA).

### 2.6. Analysis of CD47 mRNA Expression in Public Datasets

Publicly available TCGA RNA-seq data for LUAD (RRID: SCR_014514) and LUSC (RRID: SCR_003193) were accessed through the UCSC Xena Browser using the GDC TCGA Lung Adenocarcinoma and GDC TCGA Lung Squamous Cell Carcinoma cohorts. CD47 expression values were downloaded and plotted as log2(FPKM-UQ + 1).

DepMap/CCLE cell-line RNA-seq data were downloaded from the DepMap Public release (RRID: SCR_017655). CD47 expression values were then obtained from OmicsExpressionTPMLogp1HumanProteinCodingGenes.csv, in which gene-expression values are reported as log2(TPM + 1). NSCLC cell lines (*N* = 1 were identified using Model.csv metadata by retaining lung-lineage models annotated as non-small cell lung cancer, lung adenocarcinoma, lung adeno-squamous cell carcinoma, lung squamous cell carcinoma, or NSCLC. CD47 expression values (log2(TPM + 1) were plotted in GraphPad Prism as violin plots.

### 2.7. In Vivo Efficacy Studies of Bi-CD47-IT

All in vivo experiments were approved by the University of Colorado Anschutz Medical Campus Animal Care and Use Committee. All experimental animals were randomly allocated to the treatment groups. Investigators assessing tumor volume and histological findings were blinded to treatment allocation. Sample-size calculations were performed before the experiments were initiated. Sample-Size Calculations: Based on preliminary data indicating an approximately 40–50% reduction in tumor growth, a priori power analysis was performed using α = 0.05 and a statistical power of 0.80. The calculated minimum sample size was 3.48 mice per group, corresponding to a minimum of 4 mice per group. To account for potential experimental attrition and ensure adequate statistical power, the final sample sizes were set as at least *n* = 4 mice per treatment group for the humanized mouse experiments and at least *n* = 5 mice per treatment group for the NSG mouse experiments. The lung tumor volume was measured using digital vernier calipers and calculated according to the following formula: volume (mm^3^) = [length × (width × width)]/2 [22]. Mice were euthanized at their end point, defined as when the tumor volume became more than 1000 mm^3^, or body weight loss was more than 15%. Bi-CD47-IT was assessed in the following mouse models.

#### 2.7.1. Human NSCLC A549 CDX NSG Mouse Model

NSG mice (7–8 weeks old from The Jackson laboratory, Bar Harbor, ME, USA; RRID: IMSR_JAX:005557) were used for this study. Human NSCLC A549 cells (5 million) were subcutaneously injected into the dorsal flank of the NSG mice. When the tumor volume reached ~50 mm^3^ (day 4 post the A549 tumor cell injection), the tumor-bearing mice were treated with 81 µg/kg of bi-CD47-IT by daily intraperitoneally injection for 10 consecutive days. The dose schedule of bi-CD47-IT was based on our previous experience [20]. PBS was included as a vehicle treatment control. BiscFv(B6H12) (bivalent anti-human CD47 scFv, clone B6H12, without DT390) was included as a control, administered at 81 µg/kg via IP injection, once daily, for 10 consecutive days. The tumor volume was monitored twice weekly, and the tumor volume curve was recorded. When the first tumor in PBS group reached the maximum allowed volume (~1000 mm^3^) on day 40 post the A549 tumor cell injection, all tumor-bearing mice were euthanized to collect tumors for comparison of the tumor size and weight (Supplementary Figure S2A).

#### 2.7.2. Human NSCLC H1299 CDX NSG Mouse Model

NSG mice (7–8 weeks old from The Jackson laboratory) were used for this study. Human NSCLC H1299 cells (5 million) were subcutaneously injected into the dorsal flank of the NSG mice. When the tumor volume reached ~70–80 mm^3^ (day 4 post the H1299 tumor cell injection), the tumor-bearing mice were treated with 81 µg/kg of bi-CD47-IT by daily intraperitoneally injection for 10 consecutive days. PBS was included as a vehicle treatment control. BiscFv(B6H12) was included as a control. The tumor volume was monitored twice weekly, and the tumor volume curve was recorded. When the first tumor in PBS group reached the maximum allowed volume (day 28 post the H1299 tumor cell injection), all tumor-bearing mice were euthanized to collect the tumors for comparison of the tumor size and weight (Supplementary Figure S2C).

#### 2.7.3. Human NSCLC A549 CDX Humanized Mouse Model

Hu-CD34^+^ NSG-SGM3 mice (14 weeks old, RRID: MMRRC_013062-JAX) were used for this study. Human NSCLC A549 cells (5 million) were subcutaneously injected into the dorsal flank of the humanized mice. The chimerism level was confirmed by flow cytometry analysis (>25%) (Supplementary Figure S3C,D). When the tumor volume reached ~70 mm^3^ (day 5 post the A549 tumor cell injection), the tumor-bearing mice were treated with 81 µg/kg of bi-CD47-IT by daily intraperitoneal injection for 10 consecutive days. PBS treatment group was included as a vehicle treatment control. Tumor volumes were monitored twice weekly. When the first tumor in the PBS treatment group reached the maximum allowed volume, on day 33, all tumor-bearing mice were euthanized to collect the tumors for comparison of the tumor size and weight (Supplementary Figure S3A).

#### 2.7.4. Orthotopic Human NSCLC A549 CDX NSG Mouse Model

NSG mice (7–8 weeks old from The Jackson laboratory) were used for this study. Human NSCLC A549 cells (500,000) were orthotopically injected into the left lung of the NSG mice. When the orthotopic A549 NSCLC was detectable by micro-CT scan analysis (day 10 post the tumor cell injection), the tumor-bearing mice were treated with 81 µg/kg of bi-CD47-IT by daily intraperitoneal injection for 10 consecutive days. PBS treatment group was included as a vehicle treatment control. The survival curve was recorded. The tumor volume was monitored once weekly by micro-CT scan analysis. When the first tumor-bearing mouse in the PBS treatment group met the study endpoint on day 25 post the A549 tumor cell injection, two tumor-bearing mice were euthanized for gross examination and histological analysis. The remaining tumor-bearing mice were continually monitored to the study endpoint (Supplementary Figure S4A).

#### 2.7.5. Orthotopic Human NSCLC A549 CDX Humanized Mouse Model

Hu-CD34^+^ NSG-SGM3 mice (12 weeks old) were used for this study. The chimerism level was confirmed by flow cytometry analysis (>25%) (Supplementary Figure S4K–L). Human NSCLC A549 cells (500,000) were orthotopically injected into the left lung of Hu-CD34^+^ NSG-SGM3 mice. When the lung tumor was detectable by micro-CT scan analysis on day 7 post the A549 tumor cell injection, the tumor-bearing mice were treated with 81 µg/kg of bi-CD47-IT by daily intraperitoneal injection for 10 consecutive days. PBS treatment group was included as a vehicle treatment control. The survival curve was recorded. Lung tumor volume was monitored once weekly by micro-CT scan analysis. When the first tumor-bearing mouse in the PBS treatment group met the study endpoint on day 29 post the A549 tumor cell injection, two tumor-bearing mice were euthanized for gross examination and histological analysis. The remaining tumor-bearing mice were continually monitored to the study endpoint (Supplementary Figure S4C).

#### 2.7.6. Experimental Metastasis Human NSCLC A549 CDX NSG Mouse Model

NSG mice (7–8 weeks old from The Jackson laboratory) were used for this study. Human NSCLC A549 cells (2 million) were intravenously injected into the NSG mice via tail vein. Bi-CD47-IT treatment started on day 4 at 81 µg/kg, administered via intraperitoneal injection, once daily for 10 consecutive days. PBS treatment group was included as a vehicle treatment control. The survival curve was recorded. When the first tumor-bearing mouse in the PBS treatment group met the study endpoint on day 38 post the A549 tumor cell injection, two tumor-bearing mice were euthanized for gross examination and histology analyses of lungs, spleens, livers and kidneys. The remaining tumor-bearing mice were continually monitored to the study endpoint (Supplementary Figure S5A).

#### 2.7.7. Human NSCLC PDX1 (CT257-F4) NSG Mouse Model

NSG mice (7–8 weeks old from The Jackson Laboratory) were used for this study. NSCLC PDX1 tumor sample was amplified using NSG mice. The amplified fresh NSCLC PDX1 tumors were cut into small pieces and subcutaneously implanted into the dorsal flank of the experimental NSG mice. When the tumor was palpable on day 15 post the PDX1 tumor implantation, bi-CD47-IT treatment started at 81 µg/kg, administered via intraperitoneal injection, once daily for 10 consecutive days. The PBS treatment group was included as a vehicle treatment control. Tumor volumes were monitored on days 15, 25 and 44. When the first tumor in the PBS treatment group reached the maximum allowed volume, on day 44 post the PDX1 tumor implantation, all tumor-bearing mice were euthanized to collect the tumors for comparison of the tumor size and weight (Supplementary Figure S6A).

#### 2.7.8. Human NSCLC PDX2 (220900-M3) NSG Mouse Model

NSG mice (7–8 weeks old from The Jackson Laboratory) were used for this study. Human NSCLC PDX2 tumor sample was amplified using NSG mice. The amplified fresh NSCLC PDX2 tumors were cut into small pieces and subcutaneously implanted into the dorsal flank of the experimental NSG mice. When the tumor was palpable, on day 12 post the PDX2 tumor implantation, bi-CD47-IT treatment started at 81 µg/kg, administered via intraperitoneal injection, once daily for 10 consecutive days. The PBS treatment group was included as a vehicle treatment control. Tumor volumes were monitored on days 12, 22, 30 and 35. When the first tumor in the PBS treatment group reached the maximum allowed volume (day 35), all tumor-bearing mice were euthanized to collect tumors for comparison of the tumor size and weight (Supplementary Figure S6C).

#### 2.7.9. Human NSCLC PDX3 (220904-M3) NSG Mouse Model

NSG mice (7–8 weeks old from The Jackson Laboratory) were used for this study. Human NSCLC PDX3 tumor sample was amplified using NSG mice. The amplified fresh NSCLC PDX3 tumors were cut into small pieces and subcutaneously implanted into the dorsal flank of the experimental NSG mice. When the tumor was palpable, on day 9 post the PDX3 tumor implantation, bi-CD47-IT treatment started at 81 µg/kg, administered via intraperitoneal injection, once daily for 10 consecutive days. The PBS treatment group was included as a vehicle treatment control. Tumor volumes were monitored on days 9, 19, 26 and 35. When the first tumor in the PBS treatment group reached the maximum allowed volume (day 35), all tumor-bearing mice were euthanized to collect tumors for comparison of the tumor size and weight (Supplementary Figure S6E).

#### 2.7.10. In Vivo Efficacy Comparison of Bi-CD47-IT Versus Cisplatin in a Human NSCLC A549 CDX NSG Mouse Model

NSG mice (7–8 weeks old from The Jackson laboratory) were used for this study. NSCLC A549 tumor cells (5 million) were subcutaneously injected into the dorsal flank of the NSG mice. When the tumor volume reached ~50 mm^3^ (day 4 post the tumor cell injection), bi-CD47-IT treatment started at 40 μg/kg, administered via intraperitoneal injection, once daily for 10 consecutive days. Cisplatin was intraperitoneally injected at 2 mg/kg [23], once weekly with two doses total. The tumors were monitored twice weekly, and the tumor volume curve was recorded. When the first tumor in the PBS treatment group reached the maximum allowed volume (day 28), all tumor-bearing mice were euthanized to collect the tumors for comparison of the tumor size and weight (Supplementary Figure S7E).

### 2.8. Sex as a Biological Variable

Equal numbers of male and female NSG mice of the same age from the Jackson Laboratory were used for each experiment. Only female humanized Hu-CD34^+^ NSG-SGM3 mice were used in this study because human chimerism (the presence of human cells within a mouse’s system) is generally more stable in female humanized mice than in male humanized mice.

### 2.9. Histology Analysis

Murine tissues were surgically harvested from the tumor-bearing mice. The collected tissues were fixed in 10% formalin, embedded in paraffin, and subsequently sectioned. Hematoxylin and eosin (H&E) staining was performed by the University of Colorado Histology Shared Resource Core. CD47 expression was analyzed by immunohistochemistry (IHC) staining using anti-human CD47 mAb (40 µg/mL, Invitrogen), followed by a goat anti-mouse HRP-conjugated secondary antibody. Ki-67 expression was analyzed by IHC staining using an anti-Ki-67 mAb (Biocare Medical) according to the manufacturer’s protocol. Immunofluorescence (IF) staining was performed using primary mAbs (anti-human CK7, anti-human CD47, anti-human/murine CD3, anti-human/murine F4/80) and secondary antibodies: goat anti-rat IgG conjugated with Cyanine 5 (2 µg/mL, Invitrogen); goat anti-rat IgG conjugated with Alexa Fluor 488 (2 µg/mL, Abcam); and goat anti-rabbit IgG conjugated with Alexa Fluor 594 (1:1000 dilution, Abcam). All slides were incubated with the primary antibodies overnight at 4 °C, followed by incubation with the secondary antibodies for 1 h at room temperature. Nuclear staining was performed with hematoxylin for IHC, and DAPI for IF. Images were captured using an Echo Revolve Microscope (San Diego, CA, USA).

### 2.10. Statistical Analysis

Statistical analyses were performed using GraphPad Prism 11 (GraphPad Software, San Diego, CA, USA). The statistical test used for each analysis was selected based on the experimental design and is indicated in the corresponding Figure or Supplementary Figure legend. Comparisons between groups were performed using one-way or two-way analysis of variance (ANOVA), with Tukey’s or Šídák’s multiple-comparisons test, as appropriate. For repeated-measures data, two-way repeated-measures ANOVA was used, followed by the indicated multiple-comparisons test. When appropriate, data normality was assessed using the Shapiro–Wilk test, and comparisons between two groups were performed using an unpaired Welch’s *t*-test. Survival data were analyzed using the log-rank (Mantel–Cox) test. All statistical tests were two-sided, and *p* < 0.05 was considered statistically significant. Sample sizes (*n*) and the statistical tests used for individual analyses are reported in the corresponding Figure and Supplementary Figure legends.

## 3. Results

### 3.1. Bi-CD47-IT Effectively Binds to and Inhibits Human Non-Small Cell Lung Cancer (NSCLC) Cells

We first evaluated the in vitro binding activity of Alexa Fluor 488-labeled bi-CD47-IT to two representative human NSCLC cell lines by flow cytometry. We chose A549 cells with an activating KRAS G12S mutation, and inactivating STK11, KEAP1 and CDKN2A genomic changes, and H1299 cells with activating PIK3CA E545K and NRAS Q61K mutations and loss of TP53 and CDKN2A. We confirmed that both cell lines had high expression of CD47. A549 and H1299 cells express 9.7-fold and 8.2-fold more CD47, respectively, than human red blood cells (Supplementary Figure S1A). We also analyzed human CD47 protein expression in additional NSCLC cell lines (Supplementary Figure S1A). CD47 mRNA was readily detectable in TCGA LUAD and LUSC tumor samples, supporting high levels of CD47 expression in these tumors (Supplementary Figure S1C). Because TCGA bulk tumor RNA-seq includes non-malignant cells, we also evaluated CD47 expression in DepMap/CCLE NSCLC cell line models. CD47 mRNA was also detectable across NSCLC cell lines (Supplementary Figure S1D). As shown in Figure 1A, analysis of on-cell binding kinetics revealed that bi-CD47-IT bound to A549 cells with a kinetically derived affinity K_D_ of 1.27 µM and a dissociation rate of K_off_ = 0.08249 min^−1^. Similarly, bi-CD47-IT bound to H1299 cells (Figure 1C) with a closely matched kinetic K_D_ of 2.10 µM and a dissociation rate of K_off_ = 0.1055 min^−1^. To determine how receptor abundance interplays with these kinetic profiles to drive cytotoxicity, we precisely quantified absolute cell-surface CD47 expression using Molecules of Equivalent Soluble Fluorochrome (MESF)-based calibration. Both cell lines displayed a robust baseline target density: A549 cells exhibited a dense target profile with 70,472.8 MESF, and H1299 cells maintained 59,433.4 MESF (Supplementary Figure S1A). These findings indicate that the highly potent and uniform cytotoxicity observed across both cell lines is driven by comparable on-cell binding affinities combined with a consistently high absolute number of surface targets available for immunotoxin engagement. We next tested the efficacy of bi-CD47-IT against these cell lines in vitro using a CellTiter-Glo^®^ luminescent cell viability assay (Promega, Madison, WI, USA). Bivalent anti-porcine species-specific CD3 immunotoxin (pCD3-IT) was used as a negative immunotoxin control. The pCD3-IT was genetically engineered to link the same truncated diphtheria toxin, DT390, with bivalent anti-porcine CD3 epsilon scFvs [24]. Bi-CD47-IT very effectively inhibited the growth of A549 cells, with an IC_50_ value of 1.58 × 10^−11^ M (Figure 1B), and inhibited H1299 cells, with an IC_50_ of 0.97 × 10^−10^ M (Figure 1D). In contrast, pCD3-IT had no effect on cell viability. These data demonstrate that bi-CD47-IT is highly potent against NSCLC in vitro, despite its low binding affinity. We validated the CellTiter-Glo^®^ Luminescent Cell Viability Assay using direct cell counting and confirmed that the data were reliable (Supplementary Figure S1E,F). We also used Annexin-V/7-AAD staining to confirm that bi-CD47-IT induces apoptosis (Supplementary Figure S1G–J). Specifically, bi-CD47-IT internalizes into targeted cells and inhibits protein synthesis to induce tumor cell death, rather than suppressing cell growth [25].

### 3.2. Bi-CD47-IT Effectively Inhibits Tumor Growth in Two NSCLC CDX Mouse Models

We next explored the in vivo efficacy of bi-CD47-IT against NSCLC. A549 cells were subcutaneously injected into the dorsal flank of NSG (NOD scid gamma mice) mice, and once the average tumor volume reached ~50 mm^3^ (day 4 post tumor cell injection), mice were treated with 81 µg/kg of bi-CD47-IT by daily intraperitoneal injections for 10 consecutive days (Supplementary Figure S2A). The dosing schedule was based on our previous experience [20]. As shown in Figure 2A–D, compared with vehicle control, bi-CD47-IT showed significant anti-tumor activity (*p* < 0.0001), as measured by tumor volume and tumor weight. There was a significant reduction in tumor volume as early as 10 days post-treatment (day 14 post-tumor cell injection, following the 10-day bi-CD47-IT regimen), at which point four of the six tumors were no longer palpable (Figure 2B). Furthermore, tumors were completely cleared and undetectable in 50% (three of six) of the mice at the day 40 study endpoint (Figure 2C,D). We next validated these findings in an H1299 subcutaneous CDX mouse model (Supplementary Figure S2C). As shown in Figure 2E–H, bi-CD47-IT also significantly inhibited in vivo tumor growth in these cells (*p* < 0.0001). Meanwhile, we also confirmed that BiscFv(B6H12) alone is ineffective in A549 and H1299 CDX mouse models, consistent with our previous findings in the T-ALL mouse model [20]. Thus, bi-CD47-IT has potent anti-tumor activity against NSCLC in vitro and in vivo.

### 3.3. Bi-CD47-IT Demonstrates Antitumor Activity in Humanized and Orthotopic NSCLC A549 CDX Mouse Models

Immunodeficient NSG mouse models have limitations for the efficacy study, which limits the assessment of the possible interaction between bi-CD47-IT treatment versus the tumor microenvironments including human immune cells [26]. We next assessed the in vivo efficacy of bi-CD47-IT in the presence of a human immune system, using a humanized mouse model (Hu-CD34^+^-NSG-SGM3 mice) (Supplementary Figure S3A). As shown in Figure 3A–D, bi-CD47-IT significantly inhibited A549 CDX tumor growth in the presence of the human immune system (*p* < 0.0001). To mimic the advanced stages of the disease, we started the treatment later, when the tumors were slightly larger, for the bi-CD47-IT treatment in the humanized mice as compared to the NSG mice (~70 mm^3^ vs. ~50 mm^3^), and there was still substantial anti-tumor activity. No weight loss was observed, which indicates good safety of bi-CD47-IT treatment in the humanized mouse model (Supplementary Figure S3B). To evaluate whether the human immune system affects the in vivo efficacy of bi-CD47-IT, we further compared its in vivo efficacy in NSG mice versus Hu-CD34^+^ NSG-SGM3 mice head-to-head. Treatment started when tumor volumes reached ~60 mm^3^. The data demonstrated that the human immune system does not reduce the in vivo efficacy of bi-CD47-IT, as it shows comparable efficacy in both immunodeficient and humanized mouse models (Supplementary Figure S9). Tumor volumes in the PBS group of humanized mice were smaller than those in NSG mice, indicating that the human immune system itself may possess anti-tumor activity (Supplementary Figure S9). Since CD47 is also expressed on lymphocytes, monocytes, and neutrophils, we further performed a depletion profile study on them in Hu-CD34^+^ NSG-SGM3 mice. The data demonstrated that bi-CD47-IT transiently depleted lymphocytes for about a month, as we reported previously [20]. In contrast, the monocytes and neutrophils were not depleted but rather transiently expanded (Supplementary Figure S3E–G).

Because the tumor microenvironment is known to affect anti-tumor activity and orthotopic models have the potential to increase the predictive values of preclinical studies on patient response [27,28], we next tested the efficacy of bi-CD47-IT in an orthotopic model (Supplementary Figure S4A). We injected A549 cells (500,000) into the left lung of NSG mice, and when the lung tumors were detectable by micro-CT scan analysis (day 10 after tumor cell injection), the mice were treated with bi-CD47-IT, as detailed above. As shown in Figure 4D, bi-CD47-IT significantly prolonged the survival of the tumor-bearing mice, from a median survival of 25 days in the vehicle control-treated mice to 40 days in the bi-CD47-IT-treated mice. When the first tumor-bearing mouse in the control group reached the study endpoint (day 25 post the tumor cell injection), two random mice were euthanized from each group. Widespread tumor nodules were observed in the entire lungs, as well as the chests, diaphragms and abdomens of the controls (Figure 4E), as expected based on our prior experience with this cell line. In contrast, no or fewer tumor nodules in the lungs and no metastatic lesions were observed in mice treated with bi-CD47-IT. Histology analysis (H&E staining) data was consistent with the survival curve and gross examination data. Large tumor masses were observed in the lungs of the control mice, and only minimal or no residual tumors were observed in the lungs of bi-CD47-IT-treated mice, while pre-implantation lung architecture was regained (Figure 4F). The micro-CT scan image analysis data was also consistent with the survival curve, gross examination and histology analysis (H&E staining) data, with a significant reduction in tumor size in the bi-CD47-IT-treated mice (compared to the control group, *p* < 0.001) (Figure 4A–C). We further confirmed the efficacy of bi-CD47-IT in a humanized orthotopic NSCLC A549 CDX mouse model (Hu-CD34^+^-NSG-SGM3 mice) and demonstrated that bi-CD47-IT was also highly effective in the presence of a human immune system (Figure 4G–L and Supplementary Figure S4C), with no significant changes in body weight of bi-CD47-IT treated mice relative to controls (Supplementary Figure S4D). Immunohistochemistry (IHC) analyses using an anti-human CD47 mAb and an anti-Ki-67 mAb further confirmed that bi-CD47-IT is highly effective in significantly eliminating intra-tumor CD47-positive cells and this was associated with a significant reduction in Ki-67 proliferating cells in both the orthotopic NSG and humanized mouse models (Supplementary Figure S4E–H). Immunofluorescence (IF) staining using anti-human/murine CD3 and F4/80 antibodies on lung tumor cells from the orthotopic NSCLC A549 CDX humanized mouse model demonstrates that macrophages (red) and CD3^+^ T cells (green) were located in close proximity to the tumor cells in control mice, while CD3^+^ T cells were also identified in bi-CD47-IT-treated mice. Further IF staining using anti-human/murine CD3 and anti-human CD47 antibodies revealed that CD47^–^ tumor cells were surrounded by CD3^+^ T cells (purple), whereas CD47^+^ tumor cells (green) were not. Taken together, these two IF analyses indicate that bi-CD47-IT treatment did not substantially alter the CD3^+^ T-cell profile. However, the presence and distribution of CD3^+^ cells and macrophages provide supportive histological evidence but do not establish a functional immune-mediated mechanism of antitumor activity.

### 3.4. Bi-CD47-IT Was Even More Effective in an Experimental Metastasis NSCLC A549 CDX Mouse Model

To test if bi-CD47-IT is effective at preventing or treating metastatic lung cancer, the major cause of death for most lung cancer patients, A549 cells (2 million) were IV-injected into NSG mice via tail vein, and bi-CD47-IT treatment was started on day 4 (Supplementary Figure S5A). As shown in Figure 5A, bi-CD47-IT significantly prolonged the survival of the tumor-bearing mice from a median survival of 36 days in controls to 75 days in the bi-CD47-IT-treated group. On day 38, two random tumor-bearing mice were euthanized from each group. Widespread tumor nodules were observed in the representative lungs, chest wall, diaphragm and abdomen of the control mice, as expected for this cell line. In contrast, no tumor nodules were observed in the representative lungs from the bi-CD47-IT-treated group, and there were no signs of metastatic lesions (Figure 5B). Histological analysis (H&E staining) demonstrated large and invasive tumor masses in control mice and almost no tumor cells in the representative lungs of bi-CD47-IT-treated mice (Figure 5C). The spleen was slightly enlarged in controls (Figure 5D). Spleen IHC analysis using an anti-human CD47 mAb demonstrated significantly more metastatic tumor infiltration in the controls than in the bi-CD47-IT treatment group (Figure 5E,F). Lung IHC analyses using an anti-human CD47 mAb and an anti-Ki-67 mAb, as well as spleen IHC analysis using an anti-Ki-67 mAb, further confirmed that bi-CD47-IT is highly effective in the metastatic NSG mouse model (Supplementary Figure S5C–E). IF staining of lung tumor tissues and spleen sections from this metastatic A549 NSG mouse model further demonstrated that control mice exhibited numerous A549 tumors (stained with anti-human CK7/red and anti-human CD47/green), whereas these were minimal or absent in the bi-CD47-IT-treated mice (Supplementary Figure S5F–G). Gross examination and histology analysis (H&E staining) demonstrated no difference in the liver or kidney in the controls versus bi-CD47-IT-treated mice (Supplementary Figure S5H–K). These data demonstrated that bi-CD47-IT is highly effective in the experimental metastasis NSCLC CDX mouse model, which potentially has implications for the treatment of patients with advanced-stage NSCLC.

### 3.5. Bi-CD47-IT Demonstrated Potent Anti-Tumor Activity in Three Human NSCLC PDX NSG Mouse Models

Because PDXs are heterogeneous and better recapitulate lung cancer response, we further assessed the in vivo efficacy of bi-CD47-IT in three human NSCLC PDX NSG mouse models: CT-257 (with a PIK3CA E542K mutation), 220900 (with an EML4-ALK fusion), and 220904 (harboring an EGFR del exon 19 mutation with MET amplification). For PDX CT-257, PDX 220900, and PDX 220904, bi-CD47-IT treatment was started on day 15, day 12, and day 9 post-implantation, respectively (Supplementary Figure S6A,C,E). As shown in Figure 6A–F and Supplementary Figure S6J–L, bi-CD47-IT effectively inhibited tumor growth in the three PDX models. In addition, we plan to validate the efficacy of bi-CD47-IT across a broader range of NSCLC PDX models before initiating translational studies in humans.

### 3.6. Bi-CD47-IT Was More Effective than Cisplatin in an NSCLC A549 CDX Mouse Model

We hypothesized that bi-CD47-IT would be more effective than the standard-of-care chemotherapy. We chose cisplatin, a standard-of-care systemic chemotherapy for NSCLC and compared its in vivo efficacy with that of bi-CD47-IT in an A549 CDX NSG mouse model. To demonstrate a possible synergistic effect, we reduced the dose of bi-CD47-IT from normal 81 µg/kg to 40 µg/kg, administered via intraperitoneal injection once daily for 10 consecutive days. Cisplatin was administered via intraperitoneal injection at 2 mg/kg [23] once weekly for two doses (Supplementary Figure S7E). As shown in Supplementary Figure S7A–D, bi-CD47-IT, even at 50% of the normal dose, was still significantly more effective than cisplatin alone (*p* < 0.0001). However, we were unable to detect a significant difference in tumor growth between the bi-CD47-IT-only and the combination-treated group using this dosing schedule and mouse model. It might be necessary to test a different dosing schedule for the combination treatment, such as administering cisplatin first, followed by bi-CD47-IT. Experimentally, it might also be necessary to decrease the dose of bi-CD47-IT further, such as to 20 µg/kg, to demonstrate the synergistic effect compared to the bi-CD47-IT-only treatment. Body weight did not change pre- and post-treatment (Supplementary Figure S7F). With the A549 CDX model and dosing schedule tested, bi-CD47-IT exhibited greater tumor inhibition than cisplatin, supporting further evaluation of its potential efficacy relative to cisplatin.

### 3.7. Bi-CD47-IT Shows High Specificity

To evaluate the specificity of bi-CD47-IT, we performed a specificity study of bi-CD47-IT using an alternative DT390-based immunotoxin, pCD3-IT (a bivalent anti-porcine CD3 immunotoxin) [24], as a negative immunotoxin control. This control immunotoxin features the same payload but a different binding domain. Our results demonstrated that pCD3-IT did not bind to A549 cells (Supplementary Figure S8), exhibited no in vitro efficacy against A549 cells (Figure 1C), and showed no in vivo efficacy in the A549 subcutaneous CDX NSG mouse model (Supplementary Figure S9).

## 4. Discussion

CD47 is overexpressed on the surface of lung cancer cells, and on many other types of cancer cells. CD47 is also expressed on normal tissues including red blood cells and lymphocytes with a relatively lower expression level. We hypothesized that CD47 receptor could be used for targeted therapy of lung cancer and other CD47^+^ cancers. We have a diphtheria toxin-based recombinant immunotoxin development platform using a unique diphtheria toxin-resistant yeast *Pichia pastoris* expression system. This yeast expression system overcomes the expression and purification problems encountered with *E. coli*-based expression systems to deliver bi-CD47-IT with high production levels and excellent purification quality. We genetically engineered bi-CD47-IT for the targeted therapy of lung cancer and other CD47^+^ cancers. The bi-CD47-IT demonstrated “optimal” binding avidity to lung cancer and other CD47^+^ cancers, and no or weak binding to CD47^+^ normal tissues, including human red blood cells and lymphocytes, overcoming the possible toxicity to normal tissues seen with previous CD47 blockade approaches [20]. For improved solid tumor penetration and better anti-tumor efficacy, our bivalent CD47-targeting moieties could facilitate internalization by CD47 receptor crosslinking, similar to the bivalent anti-CD64-IT [29]. We speculate that the following features of bi-CD47-IT contributed to its high specificity: (1) higher binding avidity on cancer cells (due to higher CD47 expression level) than on normal cells (due to lower CD47 expression level); (2) shorter half-life than the parent mAb (~40 min vs. ~17 days); (3) lower clinical dose than the parent mAb (<20 µg/kg vs. 1–30 mg/kg). We believe that bi-CD47-IT is a promising targeted therapy drug candidate for relapsed/refractory lung cancer and other CD47^+^ cancers.

The high density of CD47 receptors on tumor cells allows for effective binding despite a lower intrinsic affinity. Bi-CD47-IT is designed with a relatively low binding affinity (e.g., K_D_ = 286.3 nM for T-ALL cells; K_D_ = 1.27 µM for A549 lung cancer cells). This “optimal” avidity ensures effective binding to high-density CD47^+^ tumor cells while showing no or weak binding to normal CD47^+^ tissues, thereby avoiding toxicities like anemia. Bi-CD47-IT is genetically engineered to contain two-tandem anti-human CD47 scFv moieties. This bivalent structure helps maintain the necessary avidity for tumor cells while the overall affinity is kept low to prevent off-target binding to RBCs. Unlike many CD47 antibodies, bi-CD47-IT does not contain an Fc region. This avoids Fc-mediated toxicities and ensures that binding is driven solely by the scFv-CD47 interaction. Internalization is a critical step for immunotoxins, as they must enter the cell to deliver their cytotoxic payload (in this case, a truncated diphtheria toxin, DT390). In immunotoxins, cytotoxicity is often related to the antigen density on the target cell membrane. A higher density can facilitate more efficient uptake. For bi-CD47-IT, the remarkably high in vitro efficacy (low IC_50_) despite the low binding affinity suggests efficient internalization and toxin delivery once bound to overexpressed CD47 on tumor surfaces.

Targeted cytotoxic cancer therapies including antibody–drug conjugates and ITs have been developed [19], and ITs in particular showed remarkable efficacy in hematologic malignancies, as evidenced by the FDA approval of Lymphir^®^ (IL-2 fusion toxin, Citius Pharmaceuticals, Inc., Cranford, NJ, USA) for cutaneous T-cell lymphoma (CTCL), Elzonris^®^ (IL-3 fusion toxin, Stemline Therapeutics, Inc., New York, NY, USA) for blastic plasmacytoid dendritic cell neoplasm (BPDCN). On-target, off-tumor toxicity is a major concern for CD47-based therapies, including bi-CD47-IT. Although we observed no toxicity of bi-CD47-IT under the conditions tested, based on in vitro binding analyses, hemagglutination assays using human red blood cells, and in vivo toxicity studies in humanized mice in both our previous [20] and current studies, these findings are insufficient to fully characterize systemic toxicity. This is particularly important given the expression of CD47 on normal hematopoietic and other cell populations and the limited information provided by body weight alone as an indicator of systemic toxicity. To address these limitations and more comprehensively evaluate the safety profile of bi-CD47-IT, studies in human CD47 knock-in mice (Applied StemCell, Milpitas, CA, USA), which broadly express human CD47 in murine tissues and organs, are currently underway.

We previously demonstrated that bi-CD47-IT was very compellingly effective against T-cell lymphoblastic leukemia (T-ALL) [20]. In this study, we further demonstrated that bi-CD47-IT was also effective against solid lung cancer. However, we noticed that bi-CD47-IT was relatively more effective against T-ALL than against solid lung cancer. We speculate that this is likely due to the inherent complexities of the tumor stroma and vasculature of solid tumors. The following observations in this study also support this rationale. We observed that the initiation date of the bi-CD47-IT treatment affected its in vivo efficacy in lung cancer. Treatment was initiated when the tumor volume reached ~70 mm^3^ in the humanized mouse model, which was later than in the NSG mouse model (~50 mm^3^). The efficacy of bi-CD47-IT is likely related to its penetration capability. We also observed that bi-CD47-IT was more effective in experimental metastasis and orthotopic mouse models than in subcutaneous mouse models. Based on these observations, we speculate that combination therapy of bi-CD47-IT with other treatments, such as surgical removal, cisplatin chemotherapy, radiotherapy, and other targeted therapy, might be more effective for lung cancer treatment. Indeed, previous studies demonstrated that both cisplatin and radiotherapy enhanced CD47 expression [30,31], while the EGFR TKI, gefitinib, or the KRAS^G12C^ inhibitor, AMG 510, reduced CD47 expression [8,10], which is consistent with the oncogenic regulation of CD47 expression by EGFR and KRAS [8,9,10]. These data provide rational therapeutic combinations to be investigated for incorporating bi-CD47-IT-targeted therapy into second and subsequent lines of therapy with standard-of-care lung cancer therapies.

The binding affinities of bi-CD47-IT to A549 and H1299 cells were relatively low (K_D_ = 1.27 µM and 2.10 µM, respectively). However, its in vitro efficacy was very impressively high (IC_50_ = 1.58 × 10^−11^ M and 0.97 × 10^−10^ M, respectively), likely due to the overexpression of CD47 on the tumor cell surface and the specific mechanism of action of bi-CD47-IT. The efficacy of bi-CD47-IT is in accordance with a large body of preclinical evidence suggesting that targeting CD47 has excellent therapeutic potential in NSCLC, particularly in EGFR-mutant tumors. This, combined with the fact that CD47 is highly expressed in a large number of NSCLCs, emphasizes the clinical implications of bi-CD47-IT, suggesting it could be broadly applicable in lung cancer patients. We speculate that diphtheria toxin-based immunotoxins, such as bi-CD47-IT, do not require high-affinity binders because of their potent, direct, enzymatic mechanism of action. Unlike conventional antibodies that rely on high affinity to initiate immune signaling, bi-CD47-IT uses two-tandem scFv moieties to create a bivalent, medium-avidity construct. This allows for high-efficiency internalization into tumor cells overexpressing CD47, while the low individual binding affinity prevents binding to normal tissues, such as RBCs, that express CD47 at lower levels. Because a single diphtheria toxin molecule reaching the cytoplasm is sufficient to cause cell death [32], high binding affinity is not required for efficient tumor-killing, allowing us to leverage this low-affinity, high-avidity approach to maximize safety.

To achieve potent and uniform cytotoxicity, an immunotoxin must successfully engage a sufficient number of target receptors. Our surface kinetic and receptor quantification assays demonstrate that both A549 and H1299 cells share highly comparable on-cell binding affinities (K_D_ of 1.27 µM and 2.10 µM, respectively). Furthermore, despite minor differences in single-molecule dissociation rates K_off_, both cell lines maintain a robust absolute CD47 surface density (~60,000 to 70,000 MESF). These findings suggest that functional cytotoxicity is driven by a receptor occupancy threshold rather than equilibrium affinity alone; because bi-CD47-IT binds with comparable kinetic affinity to a dense population of available targets on both cell lines, it readily surpasses the biological threshold required to trigger maximal downstream cell death.

We are aware that immunogenicity is always a concern with immunotoxin therapy in the setting of multiple-course treatments. However, Lymphir^@^ (IL-2 fusion toxin) and Elzonris^®^ (IL-3 fusion toxin) were approved by the FDA for multiple-course treatment. We speculate that bi-CD47-IT could also be administered for multiple-course treatment, as the same truncated diphtheria toxin, DT390, was used. We speculate that the yeast-based production systems offer some advantages over *E. coli* systems in reducing immunogenicity, primarily due to their superior protein-refolding capabilities. A designated Contract Research Organization (CRO) will comprehensively investigate the immunogenicity of bi-CD47-IT during our Good Laboratory Practice (GLP) toxicology studies.

Despite the encouraging therapeutic efficacy observed across our tested preclinical models, several critical translational hurdles remain to be addressed before clinical evaluation. First, although robust local antitumor responses were observed, the systemic toxicity profile of this diphtheria toxin-based immunotoxin has not yet been characterized in vivo in a manner sufficient to define its therapeutic window. Potential off-target tissue toxicity could therefore limit the dose that can be safely administered. Second, the potential immunogenicity of the bacterial toxin component raises the possibility of neutralizing antibody responses following repeated administration, which could reduce treatment efficacy and limit the feasibility of prolonged dosing in patients. Third, defining an optimal dosing regimen that balances antitumor potency and safety will require comprehensive pharmacokinetic and pharmacodynamic characterization. Finally, physical barriers within the tumor microenvironment, including elevated interstitial pressure and dense stromal components, may limit tumor penetration and result in heterogeneous drug distribution in human cancers, which may differ substantially from the experimental models used in the present study. Thus, these unresolved issues, including systemic toxicity, immunogenicity, dosing, and tumor penetration, represent important limitations of the current preclinical study and will need to be rigorously evaluated to establish the safety, therapeutic window, and clinical feasibility of this approach.

## 5. Conclusions

We have demonstrated that bi-CD47-IT is highly effective against NSCLC in multiple CDX and PDX mouse models, including subcutaneous, orthotopic, metastatic and humanized models. Bi-CD47-IT is a novel and promising targeted therapy for NSCLC.

## Figures and Tables

**Figure 1:**
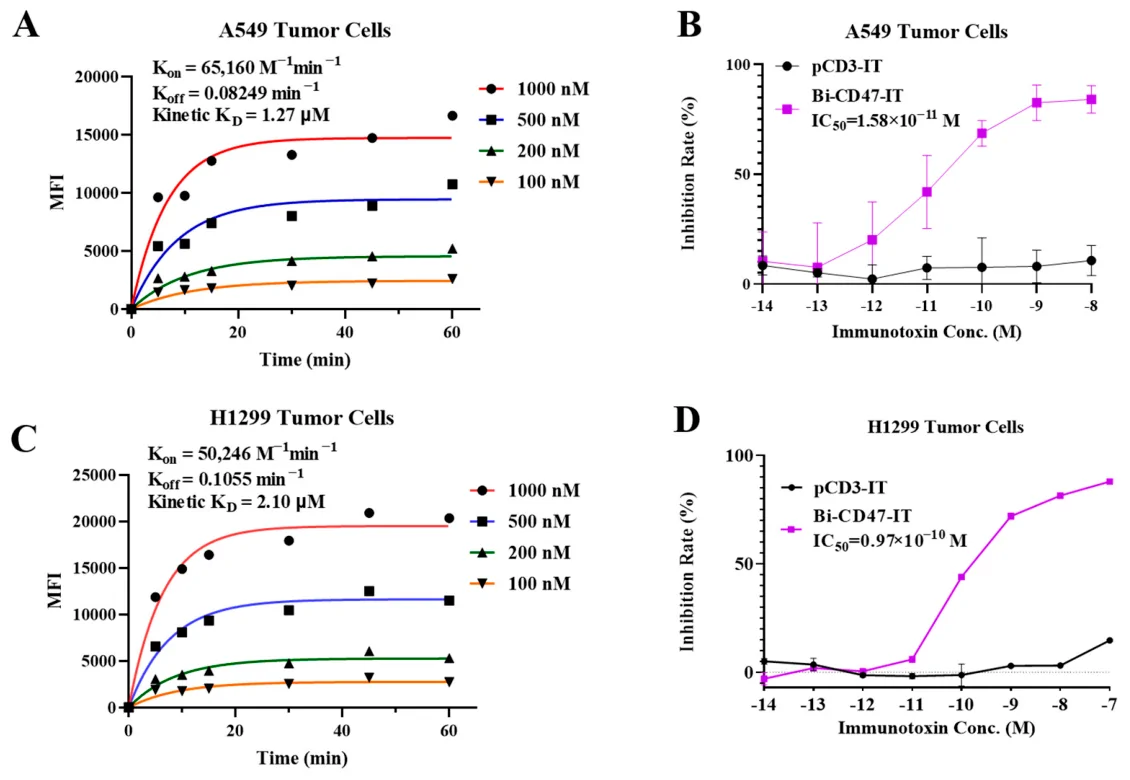
Bi-CD47-IT binds to and inhibits two non-small cell lung cancer (NSCLC) cell lines. (**A**,**C**) In vitro binding affinity analysis and K_D_ determination of Alexa Fluor 488-labeled bi-CD47-IT to human NSCLC A549 cells (**A**) and human NSCLC H1299 cells (**C**) using flow cytometry-based cell surface kinetic association and dissociation assays. FITC-anti-human CD47 mAb (B6H12) was used as a positive control, and Alexa Fluor 488-labeled isotype mouse IgG1 served as a negative control. Data are representative of three independent experiments. The K_D_ was calculated using GraphPad Prism 11 based on the equation K_D_ = k_off_/k_on_. (**B**,**D**) In vitro efficacy of bi-CD47-IT to human NSCLC A549 cells (**B**) and human NSCLC H1299 cells (**D**) determined by the CellTiter-Glo^®^ Luminescent Cell Viability Assay (purple line: bi-CD47-IT group; black line: pCD3-IT group as negative control). *Y*-axis: Percent inhibition of cell viability determined by the number of viable cells based on the quantification of ATP. *X*-axis: Immunotoxin concentration. Cycloheximide (1.25 mg/mL) was used as a positive control. The negative control wells contained cells without immunotoxin. Data is from three individual experiments. Error bars indicate SD.

**Figure 2:**
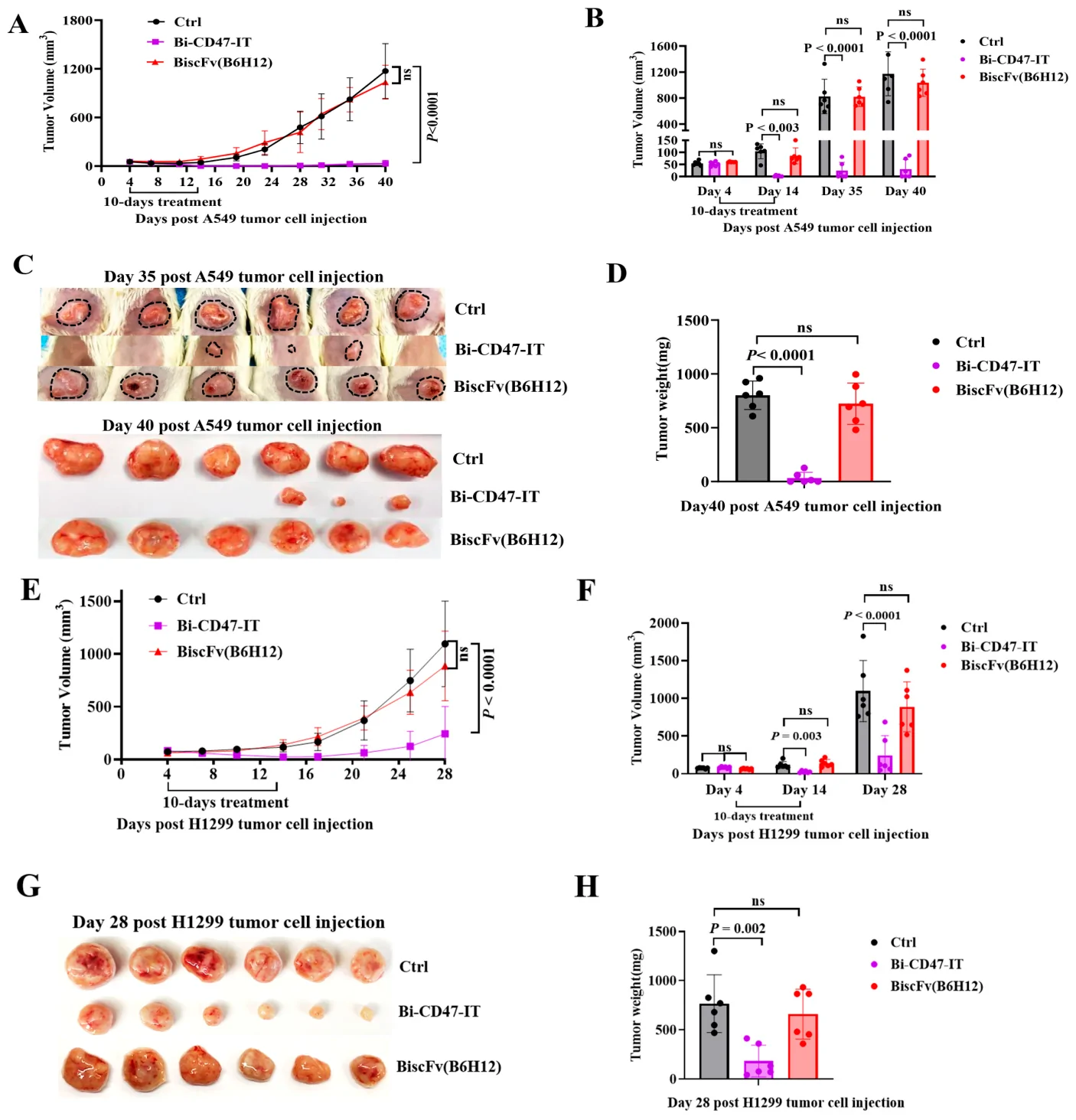
Bi-CD47-IT effectively inhibits tumor growth in two NSCLC CDX mouse models. (**A**–**D**) Human NSCLC A549 CDX mouse model (NSG mice). (**A**) A549 tumor volume growth curves. (**B**) A549 tumor volumes on days 4 (pre-treatment), 14 (post-treatment), 35, and 40 (study endpoint). (**C**) A549 tumor size on days 35 and 40. (**D**) A549 tumor weight on day 40; no visible tumors were detected in 50% (3 of 6) of the treated mice. (**E**–**H**) Human NSCLC H1299 CDX mouse model (NSG mice). (**E**) H1299 tumor volume growth curves. (**F**) H1299 tumor volumes on days 4 (pre-treatment), 14 (post-treatment), and 28 (study endpoint). (**G**) H1299 tumor size on day 28. (**H**) H1299 tumor weight on day 28. Statistical significance was calculated using two-way ANOVA followed by Tukey’s multiple comparisons test for panels A, B, E, and F, and one-way ANOVA followed by Tukey’s multiple comparisons test for panels (**D**) and (**H**) (GraphPad Prism 11; *n* = 6 per group).

**Figure 3:**
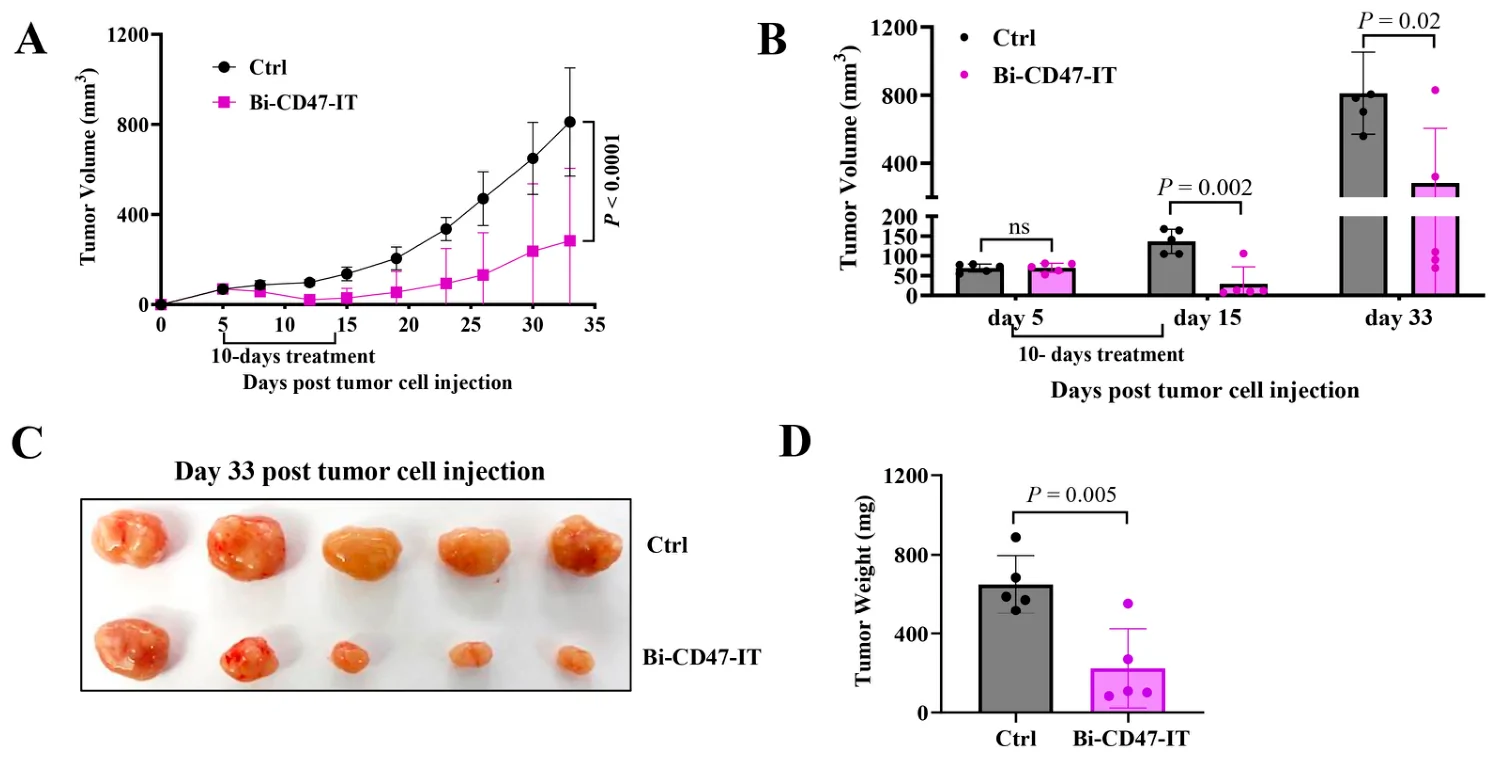
Bi-CD47-IT effectively inhibited tumor growth in a humanized NSCLC A549 CDX mouse model. Hu-CD34^+^ NSG-SGM3 mice were used for this study. (**A**) Tumor volume curves. (**B**) Tumor volume on days 5 (pre-treatment), 15 (post-treatment), and 33 (study endpoint). (**C**) Tumor size on day 33. (**D**) Tumor weight on day 33. The *p*-values were calculated using a two-way repeated-measures ANOVA followed by Tukey’s multiple comparisons test for panel (**A**), and a two-way ANOVA followed by Tukey’s multiple comparisons test for panel (**B**). Data normality was assessed using the Shapiro–Wilk test, and statistical significance was analyzed using an unpaired Welch’s *t*-test for panel (**D**) (GraphPad Prism 11; *n* = 5 per group).

**Figure 4:**
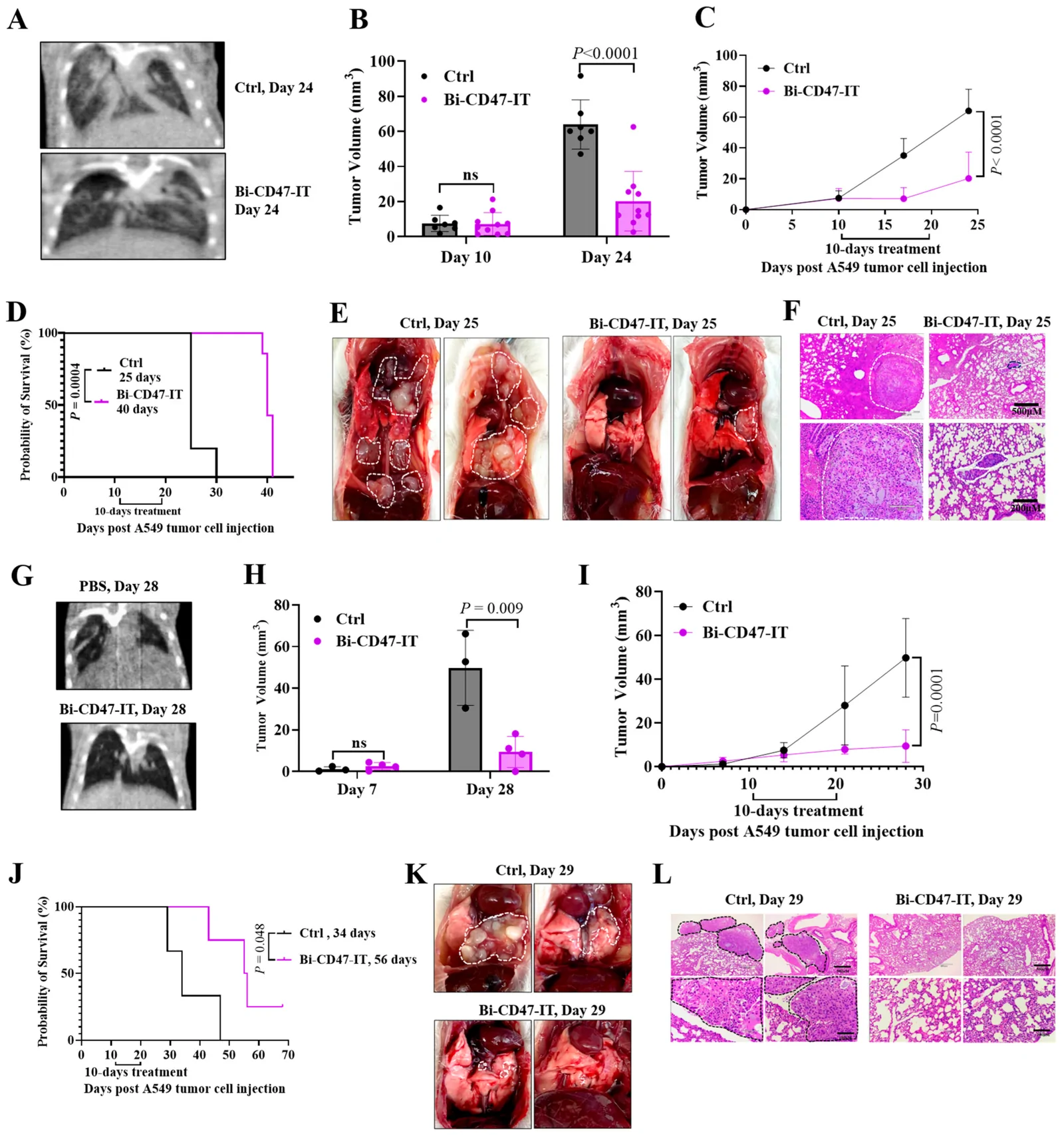
Bi-CD47-IT effectively inhibited tumor growth in orthotopic NSCLC CDX mouse models. (**A**–**F**) Orthotopic NSCLC A549 CDX mouse model (NSG mice). (**A**) Representative lungs by micro-CT scan analysis. (**B**) Tumor volumes by micro-CT scan analysis on days 10 and 24. (**C**) Tumor volume curves by micro-CT scan analysis. (**D**) Survival curves. (**E**) Gross examination of the representative lungs on day 25. (**F**) Histology analysis (H&E staining) of the representative lungs on day 25. (**G**–**L**) Humanized orthotopic NSCLC A549 CDX mouse model (Hu-CD34^+^ NSG-SGM3 mice). (**G**) Representative lungs by micro-CT scan analysis on day 28. (**H**) Tumor volumes by micro-CT scan analysis on days 7 (pre-treatment) and 28. (**I**) Tumor volume curves by micro-CT scan analysis. (**J**) Survival curves. (**K**) Gross examination of the representative lungs on day 29. (**L**) Histology analysis (H&E staining) of the representative lungs on day 29. *p*-values were calculated using GraphPad Prism 11 via a two-way ANOVA followed by Šídák’s multiple comparisons test panels (**B**,**C**,**H**,**I**) or a log-rank (Mantel–Cox) test panels (**D**,**J**). In panels (**B**) and (**C**), *n* = 7 for the control group and *n* = 10 for the bi-CD47-IT group. In panel (**D**), *n* = 5 for the control group and *n* = 7 for the bi-CD47-IT group. In panels (**H**,**I**,**J**), *n* = 3 for the control group and *n* = 4 for the bi-CD47-IT group.

**Figure 5:**
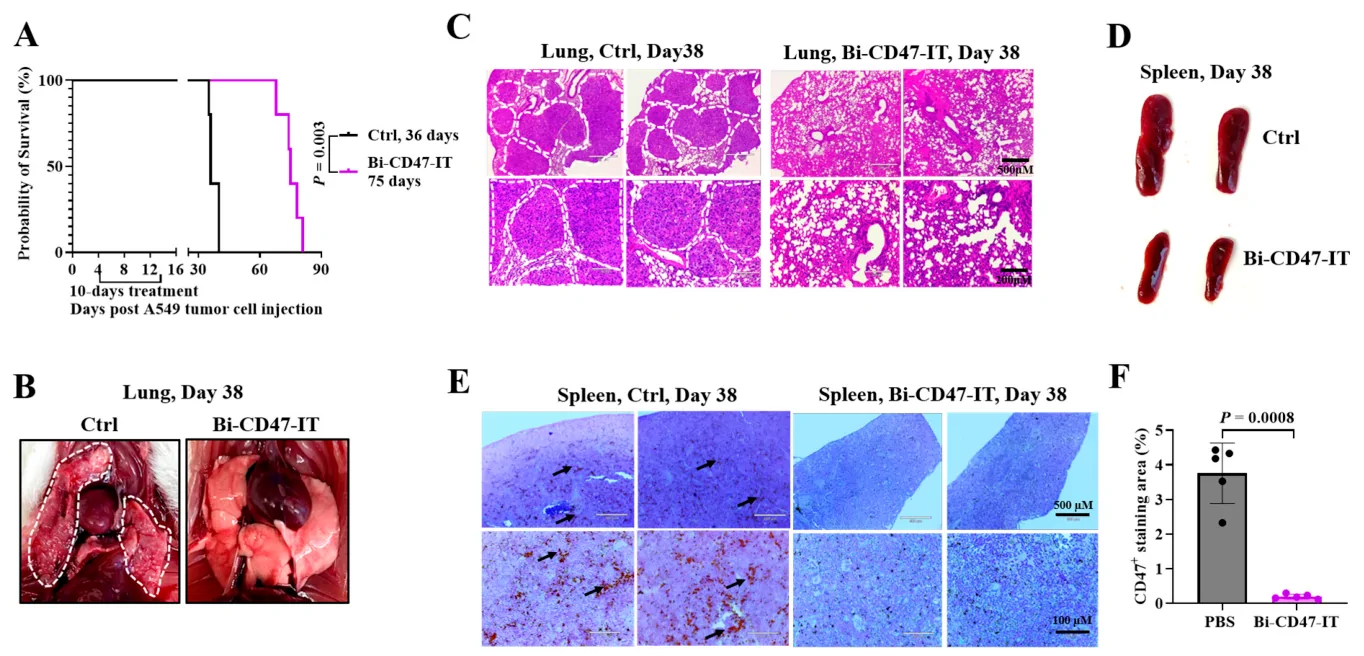
Bi-CD47-IT was even more effective in an experimental metastasis NSCLC A549 CDX mouse model. (**A**) Survival curves. (**B**) Gross examination of the representative lungs on day 38. (**C**) Histology analysis (H&E staining) of the representative lungs on day 38. (**D**) Gross examination of the representative spleens on day 38. (**E**,**F**) Immunohistochemistry (IHC) analysis of representative spleens using an anti-human CD47 mAb on day 38. The *p*-value in panel (**A**) was calculated using the Log-rank (Mantel–Cox) test, whereas for panel (**F**), data normality was tested using the Shapiro–Wilk test and significance was analyzed using an unpaired Welch’s *t*-test (GraphPad Prism 11; *n* = 5 per group).

**Figure 6:**
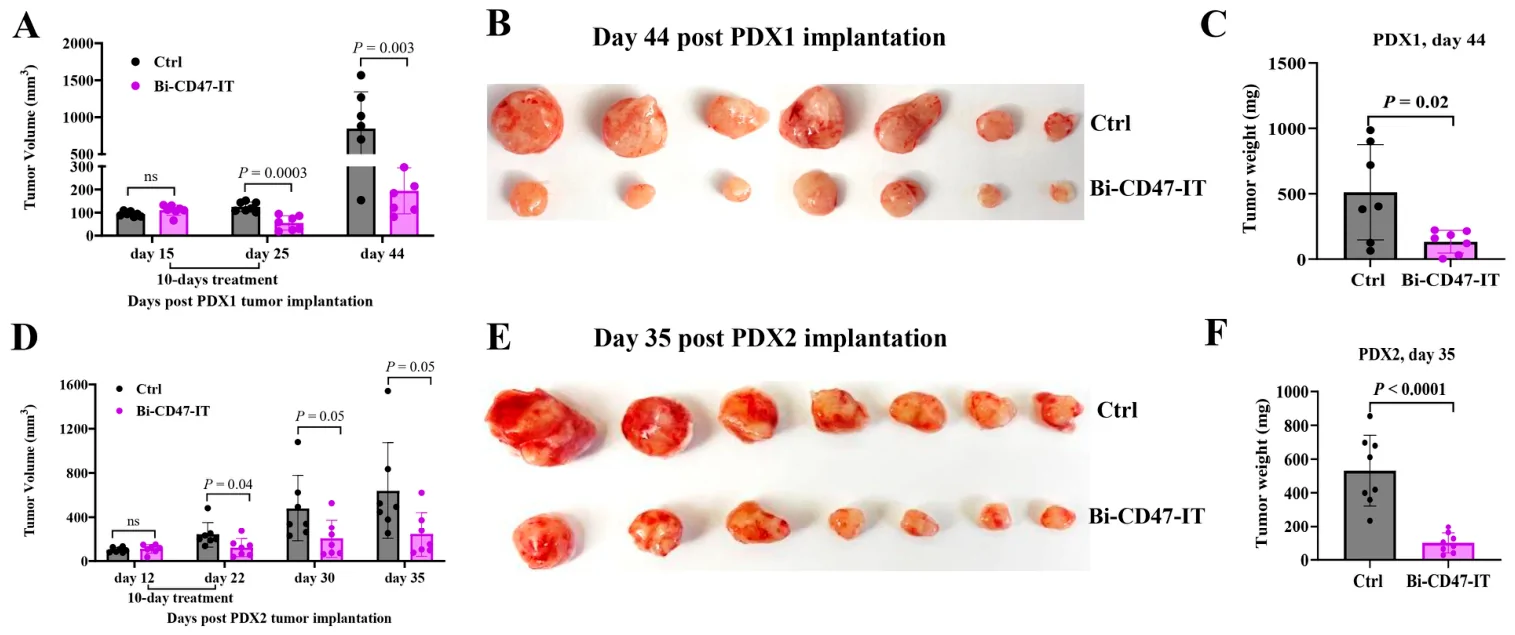
Bi-CD47-IT was highly effective in two NSCLC PDX models. (**A**–**C**) NSCLC PDX1 (CT257-F4) mouse model. (**A**) Tumor volume on days 15 (pre-treatment), 25 (post treatment) and 44 (study endpoint). (**B**) Tumor size on day 44. (**C**) Tumor weight on day 44. (**D**–**F**) NSCLC PDX2 (220900-M3) mouse model. (**D**) Tumor volume on days 12 (pre-treatment), 22 (post treatment), 30 and 35 (study endpoint). (**E**) Tumor size on day 35. (**F**) Tumor weight on day 35. The *p*-values in panels (**A**) and (**D**) were calculated using a two-way ANOVA followed by Šídák’s multiple comparisons test (GraphPad Prism 11; *n* = 7 per group). For the *p*-values in panels (**C**) and (**F**), data normality was tested using the Shapiro–Wilk test, and significance was analyzed with an unpaired Welch’s *t*-test (GraphPad Prism 11; *n* = 7 per group).

## Data Availability

The data generated in this study are available upon request from the corresponding authors.

## Supplementary Materials

The following supporting information can be downloaded at: https://www.mdpi.com/article/10.3390/biomedicines14102180/s1, Supplementary Figure S1: Supplemental information for *in vitro* binding affinity and efficacy analysis of bi-CD47-IT; Supplementary Figure S2: Supplemental information for *in vivo* efficacy studies of bi-CD47-IT in two subcutaneous NSCLC CDX mouse models; Supplementary Figure S3: Supplemental information for *in vivo* efficacy study of bi-CD47-IT in a humanized NSCLC mouse model; Supplementary Figure S4: Supplemental information for *in vivo* efficacy study of bi-CD47-IT in orthotopic NSCLC A549 CDX mouse models; Supplementary Figure S5: Supplemental information for *in vivo* efficacy study of bi-CD47-IT in an experimental lung metastasis NSCLC A549 CDX mouse model; Supplementary Figure S6: Supplemental information for the *in vivo* efficacy study of bi-CD47-IT in NSCLC PDX models; Supplementary Figure S7: Bi-CD47-IT shows superior efficacy compared to Cisplatin in an NSCLC A549 CDX *NSG* mouse model; Supplementary Figure S8: pCD3-IT does not bind to human NSCLC A549 cells *in vitro*; Supplementary Figure S9: *In vivo* efficacy comparison of bi-CD47-IT in NSG mice versus Hu-CD34^+^ NSG-SGM3 mice and *in vivo* specificity study of bi-CD47-IT using an alternative DT390-based immunotoxin, pCD3-IT (a bivalent anti-porcine CD3 immunotoxin), as a control immunotoxin; Suppementary Table S1: Antibodies used in this study; Quantification of human CD47 protein expression levels.
