# Supplementary material for: Bivalent CD47 Immunotoxin for Targeted Therapy of Lung Cancer
Source: Biomedicines. 2026 Sep 26;14(10):2180. doi: 10.3390/biomedicines14102180 (PMC13701539; doi:10.3390/biomedicines14102180)
Supplement: Supplementary file 1 [file biomedicines-14-02180-s001.zip › biomedicines-4569804-supplementary.pdf]

Supplementary Figure S1

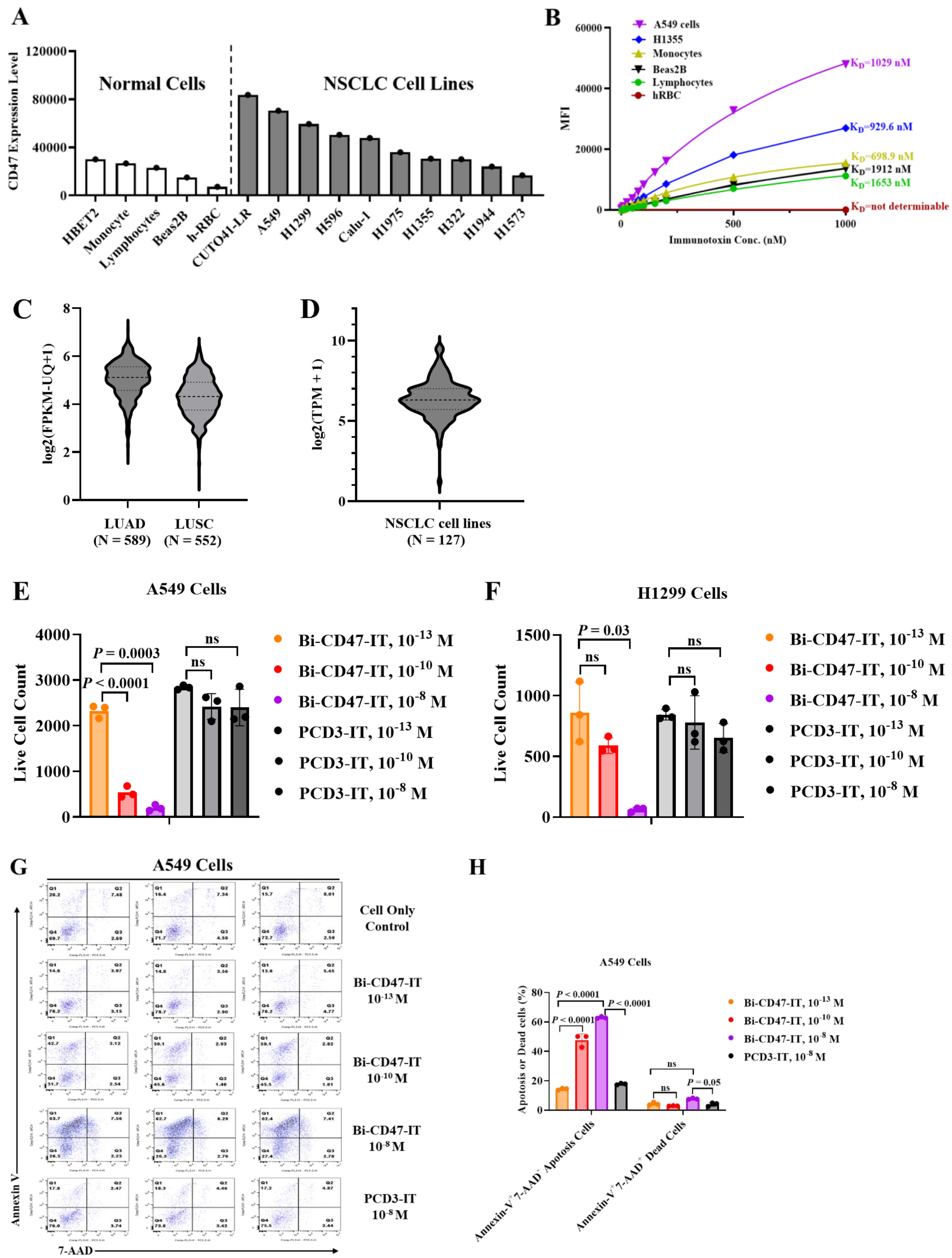

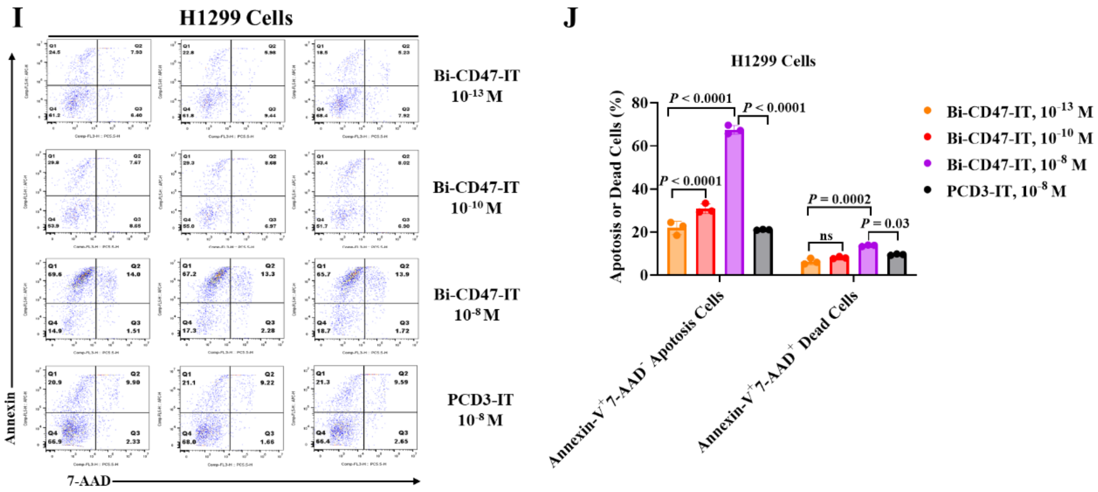

Supplementary Figure S1.

Supplemental information for *in vitro* binding affinity and efficacy analysis of bi-CD47-IT. **(A)** CD47 protein expression level of 10 human NSCLC cells (CUTO41-LR, A549, H1299, H596, Calu-1, H1975, H1355, H322, H1944, H1537) versus human red blood cells, monocytes, lymphocytes, and normal human lung epithelial cells (Beas2B and HBET2). RBC: Red blood cells. **(B)** *In vitro* binding affinity comparison of bi-CD47-IT to human NSCLC A549 cells, H1355 cells, Beas2B cells, human red blood cells, monocytes, and lymphocytes. (**C-D)** CD47 mRNA expression in lung cancer datasets and models. (**C)** CD47 expression in TCGA LUAD and LUSC tumor samples obtained from GDC TCGA cohorts through UCSC Xena were plotted as log2(FPKM-UQ + 1). LUAD: Lung Adenocarcinoma; LUSC: Lung Squamous Cell Carcinoma. (**D)** CD47 expression in DepMap/CCLE NSCLC cell-line models obtained from OmicsExpressionTPMLogp1HumanProteinCodingGenes.csv and plotted as log2(TPM + 1). NSCLC models were selected using DepMap Model.csv annotations. **(E-F)** Validation of the *in vitro* cell viability assay via direct cell counting for human NSCLC A549 **(E)** and H1299 **(F)** cells. **(G-J)** Annexin V/7-AAD staining to confirm bi-CD47-IT-induced apoptosis in A549 **(G-H)** and

H1299 **(I-J)** cells. The *p*-values in panels (**E, F, H,** and **J**) were calculated using a two-way ANOVA followed by Tukey's multiple comparisons test (GraphPad Prism 11; $n = 3$ per group).

Supplementary Figure S2

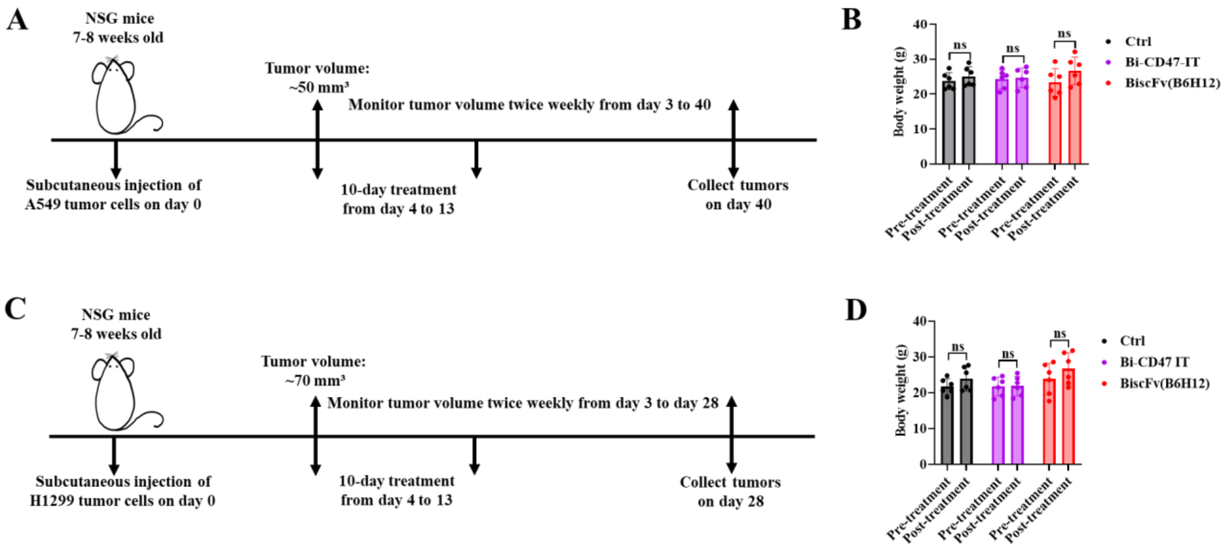

Supplementary Figure S2.

Supplemental information for *in vivo* efficacy studies of bi-CD47-IT in two subcutaneous NSCLC CDX mouse models. **(A)** Schematic diagram of the *in vivo* efficacy study of bi-CD47-IT in a human NSCLC A549 CDX mouse model. **(B)** Body weight of the A549 tumor-bearing mice on day 3 (pre-treatment) and day 14 (post-treatment). **(C)** Schematic diagram of the *in vivo* efficacy study of bi-CD47-IT in a human NSCLC H1299 CDX mouse model. **(D)** Body weight of the H1299 tumor-bearing mice on day 3 (pre-treatment) and day 14 (post-treatment). *P*-values in panels **(B)** and **(D)** were calculated using a two-way ANOVA (GraphPad Prism 11; n = 6 per group).

Supplementary Figure S3

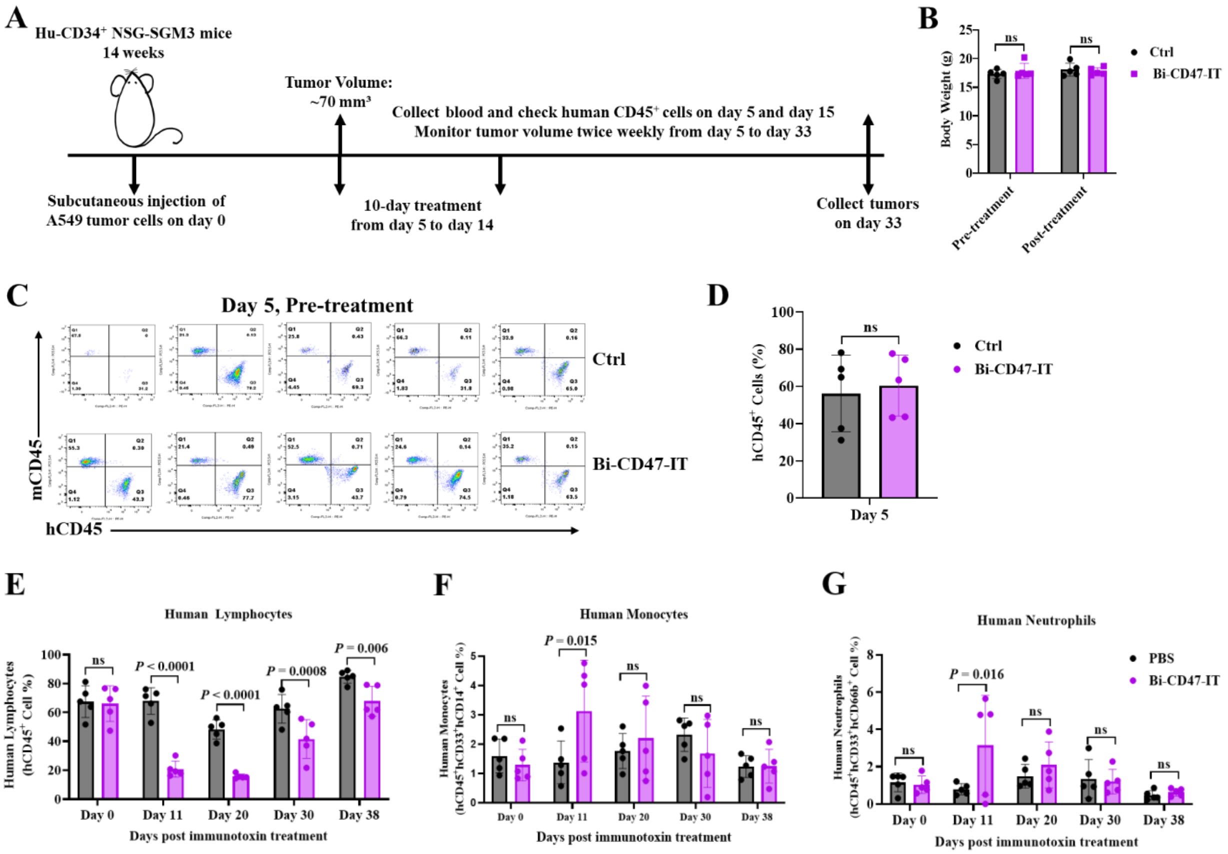

Supplementary Figure S3.

Supplemental information for *in vivo* efficacy study of bi-CD47-IT in a humanized NSCLC mouse model. **(A)** Schematic diagram of the *in vivo* efficacy study of bi-CD47-IT in a humanized NSCLC A549 CDX mouse model (Hu-CD34$^+$-NSG-SGM3 mice). **(B)** Body weight of the tumor-bearing mice on day 4 (pre-treatment) and 15 (post-treatment). **(C)** Flow cytometry analysis of the peripheral blood samples on day 5 (pre-treatment) to monitor the baseline human chimerism level. **(D)** Quantification of peripheral blood chimerism levels on day 5 (pre-treatment). mCD45, murine CD45; hCD45, human CD45. **(E-F)** Depletion profiles of bi-CD47-IT in Hu-CD34$^+$ NSG-SGM3 mice. **(E)** Human lymphocytes. The human CD45$^+$ cell percentage at Day 0 represents the baseline human chimerism level in the Hu-CD34$^+$ NSG-SGM3 mice. **(F)** Human monocytes. **(G)** Human neutrophils. The p-value in panel **(B)** was calculated using a two-way ANOVA. For panel **(D),**

data normality was verified using the Shapiro-Wilk test, and significance was analyzed with an unpaired Welch's t-test. The p-values in panels **(E, F, and G)** were calculated using a two-way repeated measures ANOVA, followed by Šídák's multiple comparisons test. All statistical analyses were performed using GraphPad Prism 11 ($n = 5$ per group).

Supplementary Figure S4

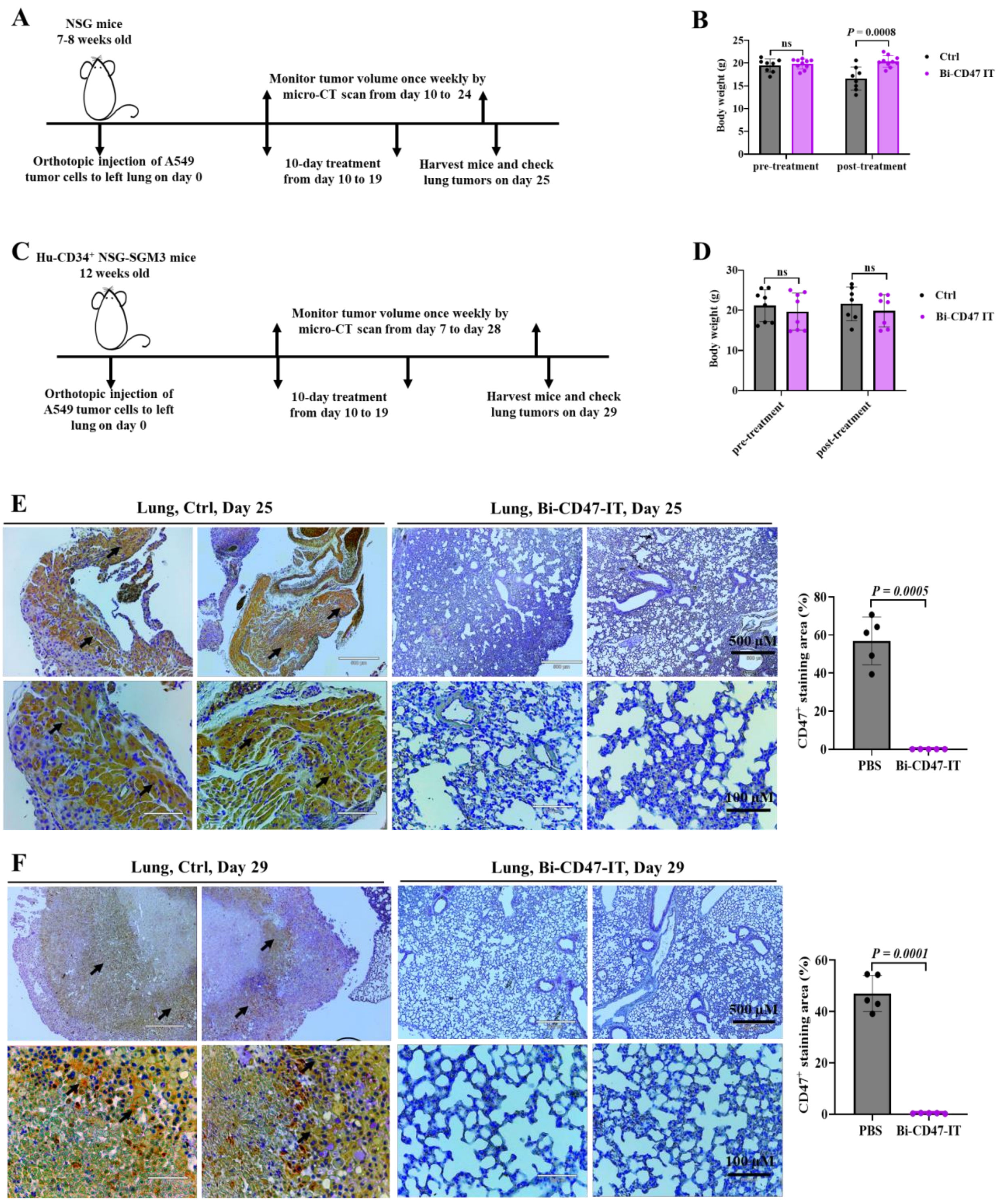

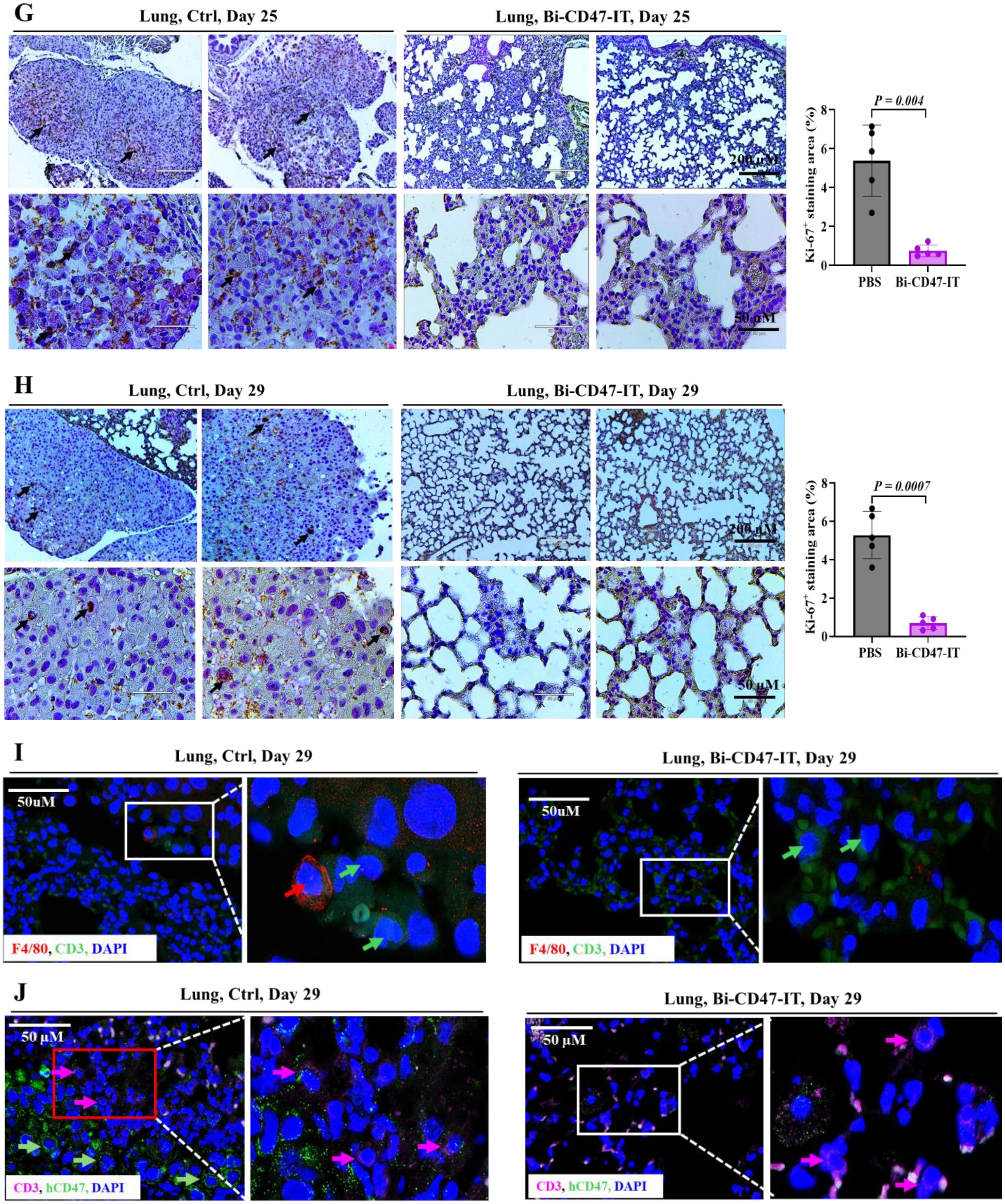
G
Lung, Ctrl, Day 25
Lung, Bi-CD47-IT, Day 25
200 uM
50 uM
P = 0.004
Ki-67+ staining area (%)
PBS
Bi-CD47-IT
H
Lung, Ctrl, Day 29
Lung, Bi-CD47-IT, Day 29
200 uM
50 uM
P = 0.0007
Ki-67+ staining area (%)
PBS
Bi-CD47-IT
I
Lung, Ctrl, Day 29
Lung, Bi-CD47-IT, Day 29
50uM
F4/80, CD3, DAPI
J
Lung, Ctrl, Day 29
Lung, Bi-CD47-IT, Day 29
50 μM
CD3, hCD47, DAPI

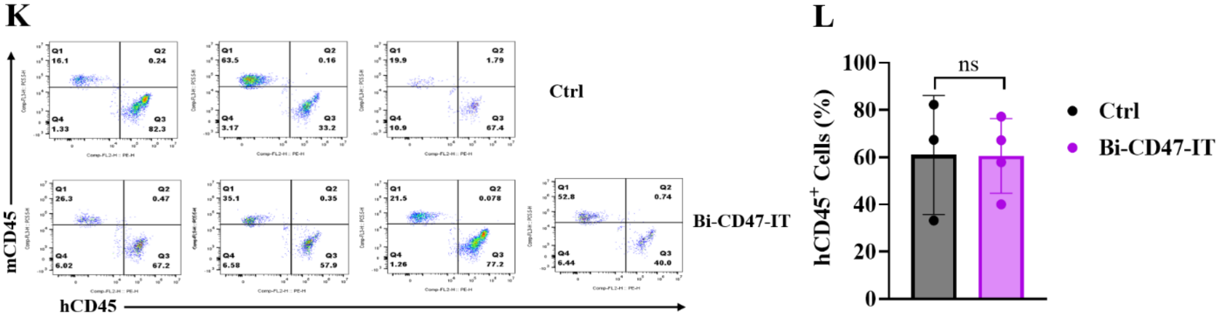

Supplementary Figure S4.

Supplemental information for *in vivo* efficacy study of bi-CD47-IT in orthotopic NSCLC A549 CDX mouse models. **(A)** Schematic diagram of the experimental design for the *in vivo* efficacy study of bi-CD47-IT in an orthotopic NSCLC A549 CDX mouse model using *NSG* mice. **(B)** Body weight of tumor-bearing mice on day 9 (pre-treatment) and day 20 (post-treatment) in the *NSG* model. **(C)** Schematic diagram of the *in vivo* efficacy study of bi-CD47-IT in a humanized orthotopic NSCLC A549 CDX mouse model (Hu-CD34+-NSG-SGM3 mice). **(D)** Body weight of tumor-bearing mice on day 9 (pre-treatment) and day 20 (post-treatment) in the humanized model. **(E)** Immunohistochemistry (IHC) staining using an anti-human CD47 mAb on lung tissues from an orthotopic NSCLC A549 CDX *NSG* mouse model. Brown positive staining (black arrows) was detected on most lung tissues of the vehicle control group but was absent in the lung tissues of the bi-CD47-IT treatment group. **(F)** IHC staining using an anti-human CD47 mAb on lung tissues from an orthotopic NSCLC A549 CDX humanized mouse model. Brown positive staining (black arrows) was detected on most lung tissues of the vehicle control group but was absent in the lung tissues of the bi-CD47-IT treatment group. **(G)** IHC staining using an anti-Ki-67 mAb on lung tumor cells from an orthotopic NSCLC A549 CDX *NSG* mouse model. Brown positive Ki-67 staining (black arrows) was detected in the nuclei of some tumor cells in the vehicle control group but was absent in the lung tissues of the bi-CD47-IT treatment group. This suggests that

tumor cells in the control group are actively proliferating. **(H**) IHC staining using an anti-Ki-67 mAb on lung tumor cells from an orthotopic NSCLC A549 CDX humanized mouse model. Brown, positive Ki-67 staining nuclei (black arrows) were detected in some tumor cells in the vehicle control group but were absent in the lung tissues of the bi-CD47-IT treatment group. This suggests that tumor cells in the control group are actively proliferating. **(I**) IF staining using anti-human/murine CD3 mAb and anti-human/murine F4/80 mAb on lung tumor cells from an orthotopic NSCLC A549 CDX humanized mouse model. Both macrophages (red) and $CD3^+$ T cells (green) were detected close to the tumor cells in control mice, while $CD3^+$ T cells were also identified in bi-CD47-IT treated mice. **(J**) IF staining using anti-human/murine CD3 mAb and anti-human CD47 mAb on lung tumor cells from an orthotopic NSCLC A549 CDX humanized mouse model. $CD47^-$ tumor cells were surrounded by $CD3^+$ T cells (purple), whereas $CD47^+$ tumor cells (green) were not. **(K-L)** Baseline human chimerism levels in Hu-$CD34^+$ NSG-SGM3 mice. **(K)** Flow cytometry plots showing human $CD45^+$ cell engraftment in peripheral blood. **(L)** Quantification of human chimerism levels between the two experimental groups (Control group and Bi-CD47-IT group). The p-values in panels **B** and **D** were calculated using a two-way ANOVA. For panel **B,** n = 8 for the control group and n = 10 for the bi-CD47-IT group. For panel **D**, n = 8 for the pre-treatment group and n = 7 for the post-treatment group. For panels **E, F, G, and H**, data normality was verified using the Shapiro–Wilk test, and statistical significance was analyzed using an unpaired Welch's t-test (n = 5 per group). For panel **L,** data normality was verified using the Shapiro–Wilk test, and statistical significance was analyzed using an unpaired Welch's t-test (n = 3 for the control group and n = 4 for the bi-CD47-IT group). All statistical analyses were performed using GraphPad Prism 11.

Supplementary Figure S5

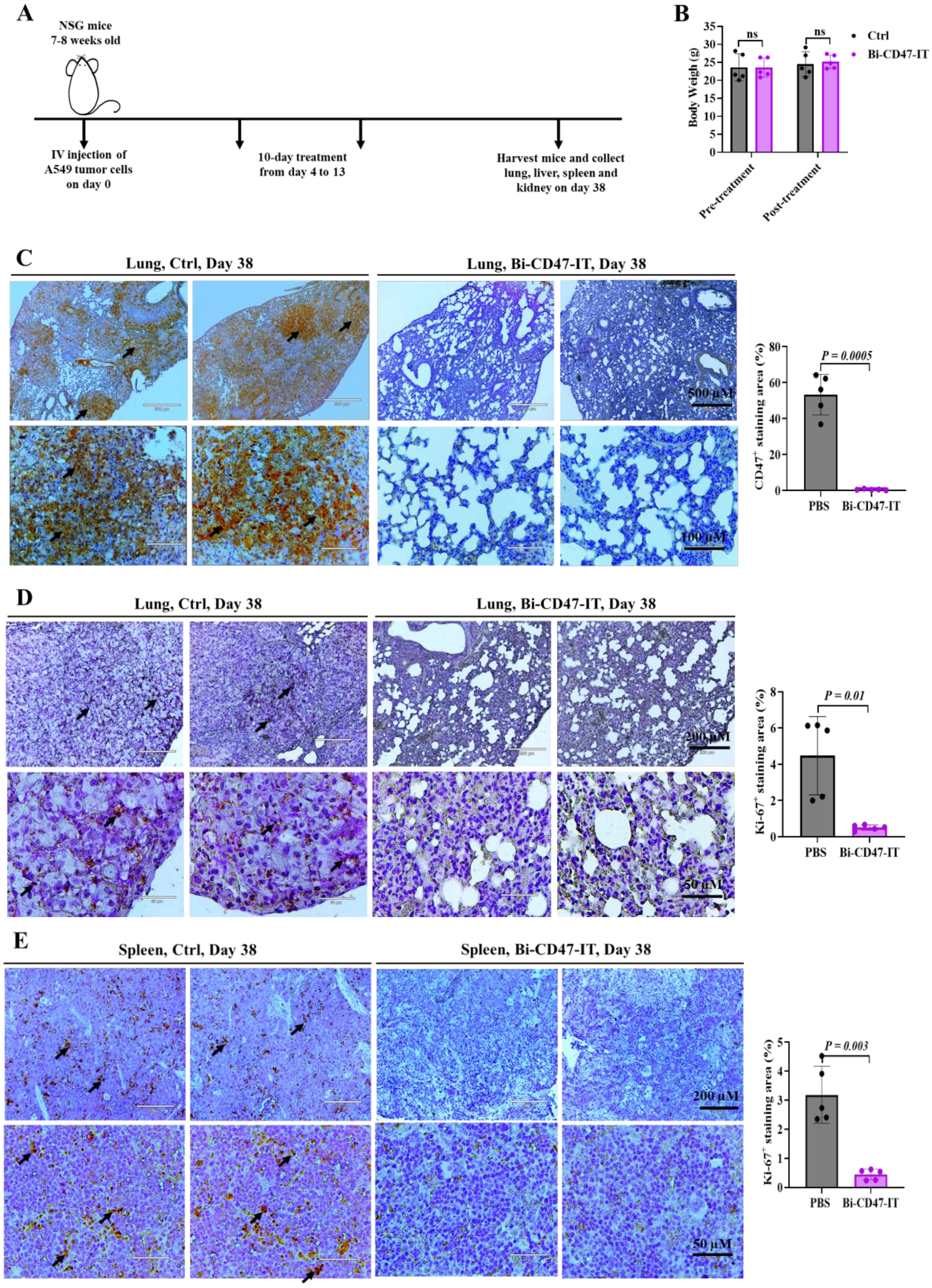

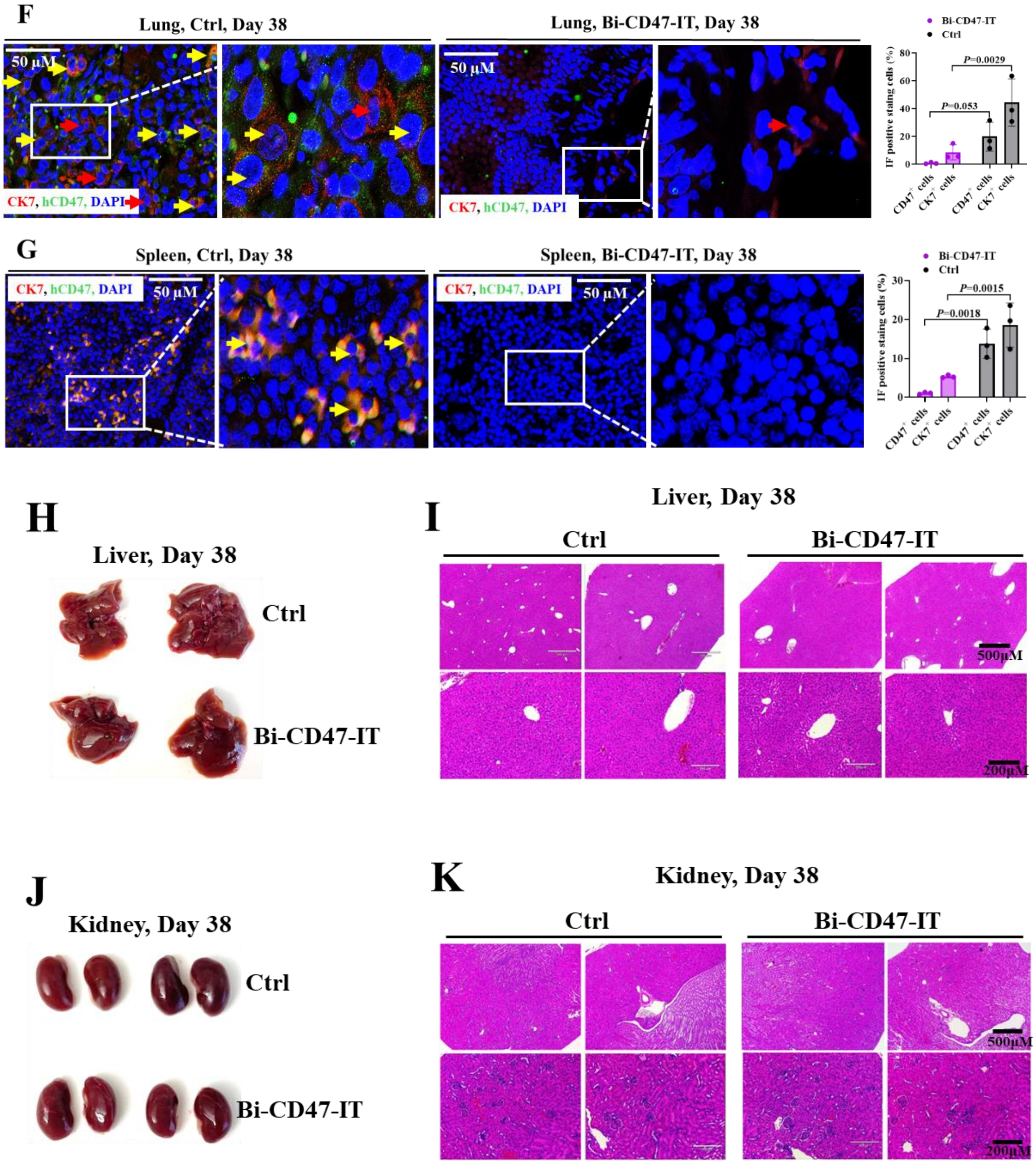

Supplementary Figure S5.

Supplemental information for *in vivo* efficacy study of bi-CD47-IT in an experimental lung metastasis NSCLC A549 CDX mouse model. **(A)** Schematic diagram of the *in vivo* efficacy study

of bi-CD47-IT in an experimental metastasis NSCLC A549 CDX *NSG* mouse model. **(B)** Body weight of the tumor-bearing mice on day 3 (pre-treatment) and day 14 (post-treatment) in the experimental metastasis model. **(C)** IHC staining using an anti-human CD47 mAb on lung tissues from a metastatic A549 NSCLC CDX *NSG* mouse model. Brown positive staining (black arrows) was detected in the vehicle control group but was absent in the bi-CD47-IT treatment group. **(D)** IHC staining using an anti-Ki-67 mAb on lung tissues from an A549 NSCLC CDX *NSG* mouse model of metastasis. Brown positive Ki-67 staining (black arrows) was detected in the nuclei of tumor cells in the vehicle control group but was absent in the lung tissues of the bi-CD47-IT treatment group. This suggests that tumor cells in the control group are actively proliferating. **(E)** IHC staining using an anti-Ki-67 mAb on spleen sections from an A549 NSCLC metastasis CDX *NSG* mouse model. Brown, positive Ki-67 staining (black arrows) was detected in some splenic cell nuclei of the vehicle control group; however, it was absent in the spleen of the bi-CD47-IT treatment group. This suggests that the tumor cells in the control group are actively proliferating. **(F)** IF staining using anti-human CK7 and anti-human CD47 mAbs was performed on lung tumor cells from a metastatic NSCLC A549 *NSG* mouse model. A549 tumor cells were stained for both CK7 (red) and hCD47 (green). High levels of staining were observed in the control mice, but lower levels were observed in the bi-CD47-IT treated mice. **(G)** Representative IF staining using anti-human CK7 (red) and anti-human CD47 (green) mAbs on spleen sections from *NSG* mice injected with A549 cells. CK7+/hCD47+ tumor cells are detected in the spleens of control mice, while they are absent in the bi-CD47-IT-treated mice. **(H)** Gross examination of two representative livers on day 38 post-tumor cell injection. **(I)** H&E staining of two representative livers on day 38 post-tumor cell injection. **(J)** Gross examination of two representative kidneys on day 38 post-tumor cell injection. **(K)** H&E staining of two representative kidneys on day 38 post-

tumor cell injection. The p-value in panel **(B)** was calculated using a two-way ANOVA. For the p-values in panels **(C, D, and E),** data normality was tested using the Shapiro-Wilk test, and significance was analyzed with an unpaired Welch's *t*-test (n = 5 per group). The p-values in panels **(F and G)** were calculated using a two-way ANOVA, followed by an uncorrected Fisher's LSD test (n = 3) per group). All statistical analyses were performed using GraphPad Prism 11.

Supplementary Figure S6

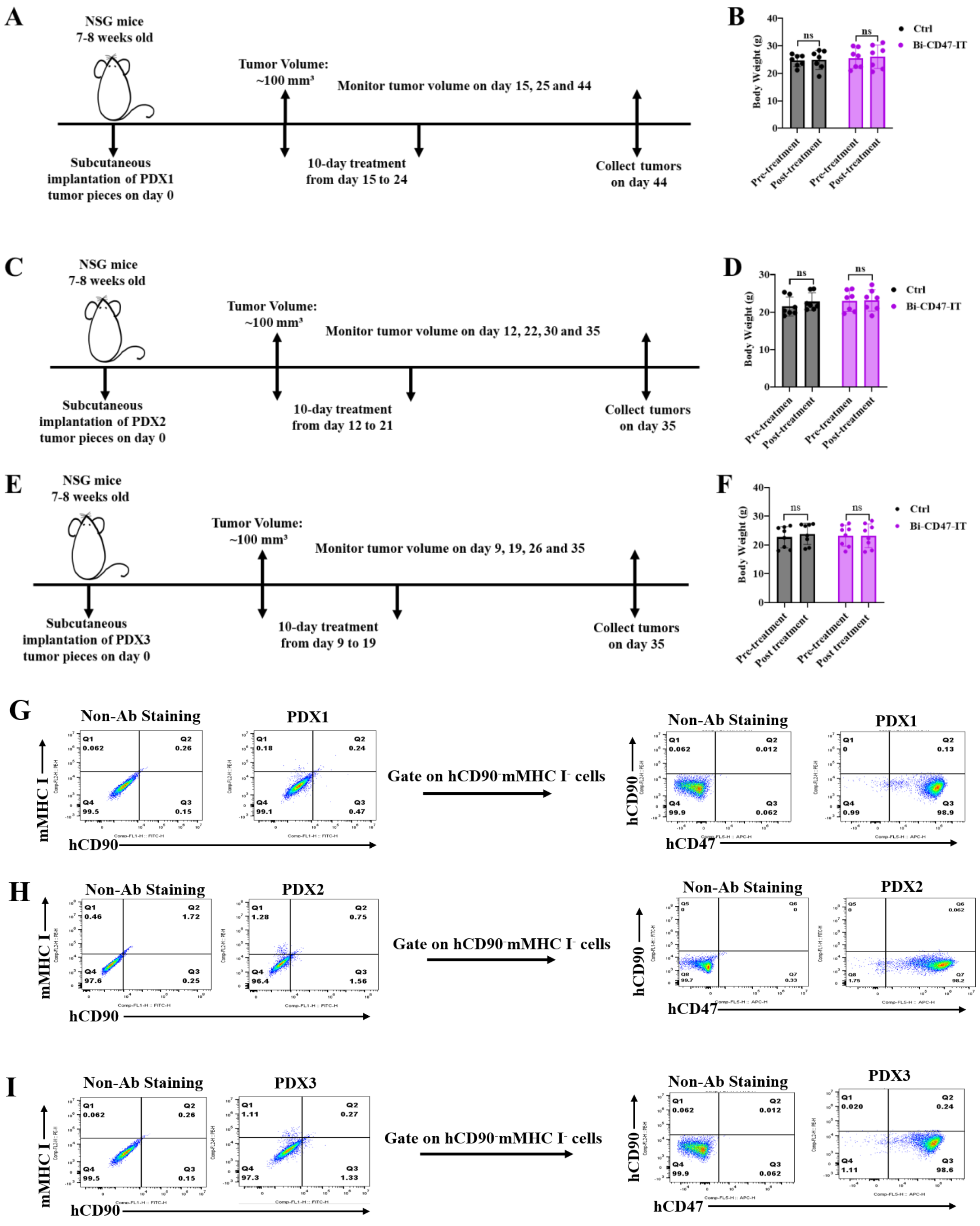

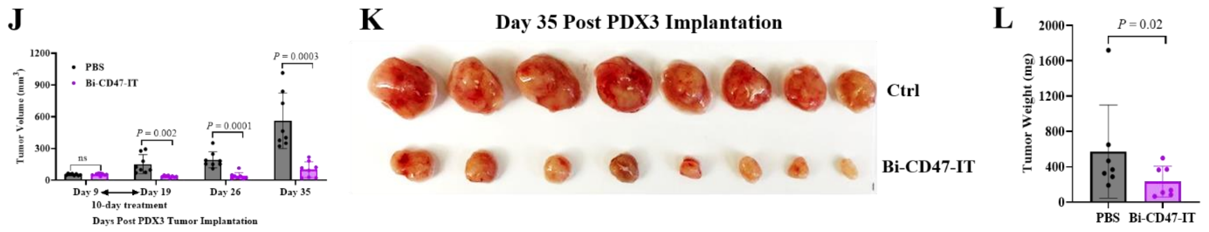

Supplementary Figure S6.

Supplemental information for the *in vivo* efficacy study of bi-CD47-IT in NSCLC PDX models. **(A)** Schematic diagram of the *in vivo* efficacy study of bi-CD47-IT in the NSCLC PDX1 (CT257-F4) mouse model. **(B)** Body weight of tumor-bearing mice on day 14 (pre-treatment) and day 25 (post-treatment) in the PDX1 mouse model. **(C)** Schematic diagram of the in vivo efficacy study of bi-CD47-IT in the NSCLC PDX2 (220900-M3) mouse model. **(D)** Body weight of tumor-bearing mice on day 11 (pre-treatment) and day 22 (post-treatment) in the PDX2 mouse model. **(E)** Schematic diagram of the *in vivo* efficacy study of bi-CD47-IT in the NSCLC PDX3 (220904-M3) mouse model. **(F)** Body weight of tumor-bearing mice on day 9 (pre-treatment) and day 19 (post-treatment) in the PDX3 mouse model. **(G-I)** NSCLC PDX1, PDX2 and PDX3 are all CD47 positive. Flow cytometry binding analysis of NSCLC PDX1 **(G)**, PDX2 **(H)** and PDX3 **(I)** to human CD47 mAb. **(J-L)** Bi-CD47-IT was also highly effective in NSCLC PDX3 (220904-M3) mouse model. **(J)** Tumor volume on day 9 (pre-treatment), 19 (post treatment), 26 and 35 (study endpoint). **(K)** Tumor size on day 35. **(L)** Tumor weighs on day 35. The *p*-values in panels **B, D, and F** were calculated using a two-way ANOVA (n = 7 per group). The *p*-values in panel **J** were determined using a two-way ANOVA followed by Šídák's multiple comparisons test (n = 8 per group). For panel **L**, data normality was assessed using the Shapiro-Wilk test, and statistical significance was analyzed with an unpaired Welch's *t*-test (n = 8 per group). All statistical analyses were performed using GraphPad Prism 11.

Supplementary Figure S7

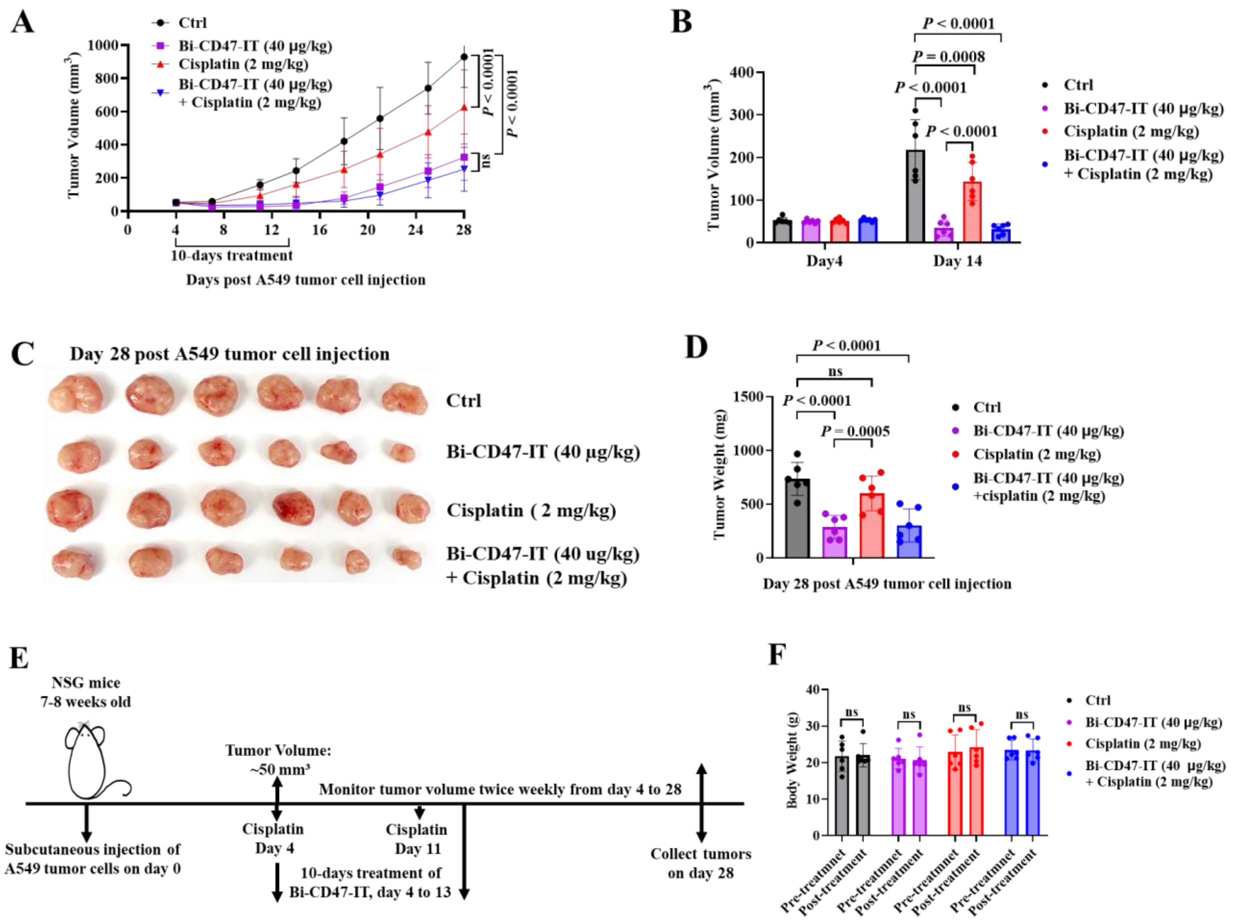

Supplementary Figure S7.

Bi-CD47-IT shows superior efficacy compared to Cisplatin in an NSCLC A549 CDX *NSG* mouse model. **(A)** Tumor volume curves. **(B)** Tumor volume on day 4 (pre-treatment) and day 14 (post-treatment). **(C)** Tumor size. **(D)** Tumor weight. **(E)** Schematic diagram of the *in vivo* efficacy study of Bi-CD47-IT versus Cisplatin in an NSCLC A549 CDX mouse model. **(F)** Body weight of the tumor-bearing mice on day 3 (pre-treatment) and 14 (post-treatment). P-values for panel **A** were calculated using a two-way repeated-measures ANOVA followed by Tukey's multiple comparisons test. For panels **B and D**, a standard two-way ANOVA with Tukey's multiple

comparisons test was used, while panel **F** was analyzed using a two-way ANOVA alone. All statistical analyses were performed using GraphPad Prism 11 (n = 6 per group).

Supplementary Figure S8

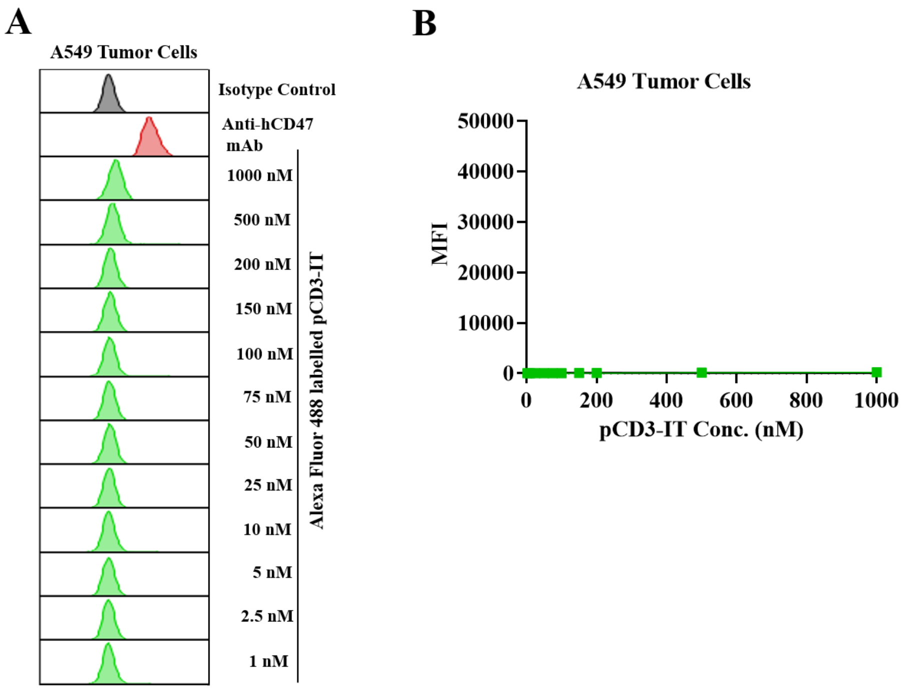

Supplementary Figure S8.

pCD3-IT does not bind to human NSCLC A549 cells *in vitro*. **(A)** *In vitro* binding of the Alexa Fluor 488-labeled pCD3-IT to human NSCLC A549 cells by flow cytometry analysis. FITC-anti-human CD47 mAb (B6H12) was used as a positive control, and Alexa Fluor 488-labeled isotype mouse IgG1 served as a negative control. The data are representative of three individual experiments. **(B)** K_D determination of pCD3-IT to human NSCLC A549 cells using flow cytometry and nonlinear least-squares fitting. The mean fluorescence intensity (MFI) was plotted over a wide range of Alexa Fluor 488-labeled pCD3-IT concentrations. Nonlinear regression was based on the equation $Y = Bmax \times X / (K_D + X)$, where Y = MFI at the given Alexa Fluor 488-labeled pCD3-IT after subtracting the background, X = Alexa Fluor 488-labeled pCD3-IT concentration, and Bmax = the maximum specific binding in the same units as Y.

Supplementary Figure S9

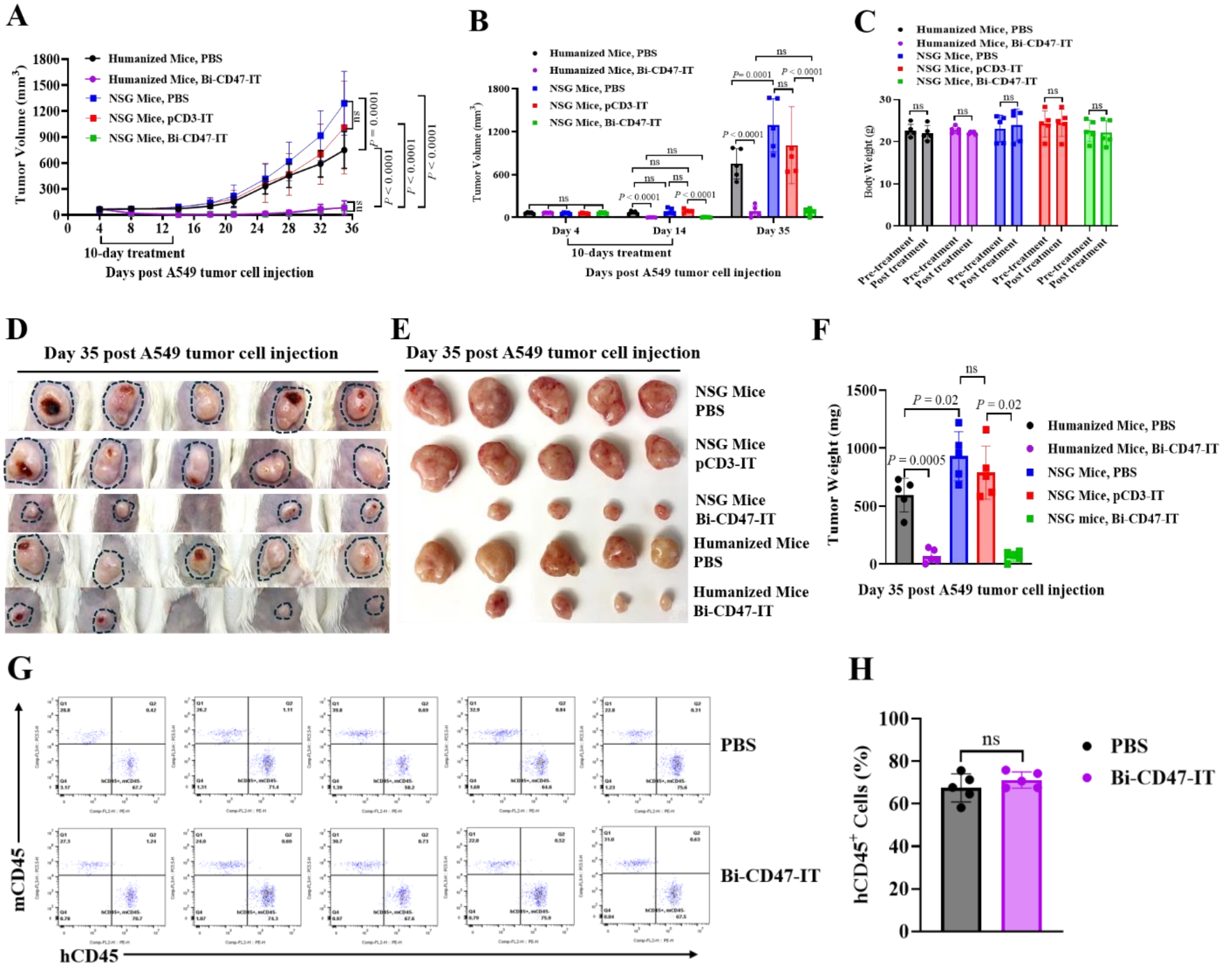

Supplementary Figure S9.

In vivo efficacy comparison of bi-CD47-IT in *NSG* mice versus Hu-CD34⁺ NSG-SGM3 mice and *in vivo* specificity study of bi-CD47-IT using an alternative DT390-based immunotoxin, pCD3-IT (a bivalent anti-porcine CD3 immunotoxin), as a control immunotoxin. This control immunotoxin features the same payload but a different binding domain. Immunotoxin treatment started when tumor volumes reached ~60 mm³ on day 4 via intraperitoneal (IP) injection at 81 µg/kg, administered once daily for 10 consecutive days. **(A)** Tumor volume curves. **(B)** Tumor volumes on days 4 (pre-treatment), 14 (post-treatment), and 35 (study endpoint). **(C)** Body weights on day

4 (pre-treatment) and day 14 (post-treatment). **(D)** Tumor volumes on day 35. **(E)** Tumor size on day 35. **(F)** Tumor weights on day 35. **(G-H)** Baseline human chimerism levels in Hu-CD34$^+$ NSG-SGM3 mice. **(G)** Flow cytometry plots showing human CD45$^+$ cell engraftment in peripheral blood. **(H)** Quantification of human chimerism levels between the two experimental groups (PBS group and Bi-CD47-IT group). The p-values in panels **A and B** were calculated using a two-way repeated measures ANOVA followed by Tukey's multiple comparisons test. The p-values in panel **C** were calculated using a two-way ANOVA, and those in panel **F** were calculated using a one-way ANOVA followed by Tukey’s multiple comparisons test. For the p-value in panel **H**, data normality was verified using the Shapiro-Wilk test, and significance was analyzed with an unpaired Welch's t-test. All statistical analyses were performed using GraphPad Prism 11 (n = 5 per group).

Supplementary Table S1. Antibodies used in this study

Antibody Name	Clone	Source	Cat#	RRID
Mouse Anti-mouse MHC Class I	2G5	Bio-Rad	MCA2189	AB_1101889
APC Anti-human CD47 Antibody	CC2C6	Biolegend	323124	AB_2716203
FITC Mouse Anti-human CD47 mAb	B6H12	BD Biosciences	556045	AB_396316
PerCP/Cy5.5 Anti-mouse CD45 mAb	C363-16A	Biolegend	103314	AB_2284707
PE Anti-human CD45 Antibody	HI30	Biolegend	304058	AB_2564156
FITC Mouse Anti-Human CD90	Clone 5E10	BD Biosciences	555595	AB_395969
Anti-human CD47 Antibody	B6H12	Invitrogen	14-0479-82	AB_837143
Anti-human Ki-67 Antibody	DVB-2	Biocare Medical	PPM 240 DS AA	AB_2721189
Anti-human CK7 mAb	D1E4	Cell Signaling	4465	AB_11178382
Anti-human/murine CD3 mAb	17A2	Invitrogen	14-0032-82	AB_467053
Anti-human/murine F4/80 mAb	Q61549	Proteintech	29414-1-AP	AB_2918300
Goat anti-mouse HRP-conjugated secondary antibody		Vector	MP-7452	AB_2744550
Goat anti-rat IgG conjugated with Cyanine 5		Invitrogen	A10525	AB_2534034
Goat anti-rat IgG conjugated with Alexa Fluor 488		Abcam	Ab150157	AB_2722511
Goat anti-rabbit IgG conjugated with Alexa Fluor 594		Abcam	Ab150080	AB_2650602
eBioscience™ 7-AAD Viability Staining Solution		ThermoFisher Scientific	00-6993-50	AB_2869408

APC Annexin V	Biolegend	640919	AB_2616657

Quantification of Human CD47 Protein Expression Levels

Antibody titration and quantification of cell-surface CD47 expression were performed on NSCLC cell lines, normal human red blood cells (RBCs), monocytes, lymphocytes, and lung epithelial cells. The anti-human CD47 monoclonal antibody (mAb, clone B6H12) was conjugated to Alexa Fluor® 488 as previously described. The conjugated antibody was titrated at concentrations of 1000, 500, 200, 100, 50, 25, 12, 1.5 nM to determine the concentration required for saturation. A saturating concentration of 1000 nM was identified and used for subsequent CD47 staining.

CD47 expression was quantified using the Quantum™ Alexa Fluor® 488 MESF kit (Bangs Laboratories, Fishers, IN, USA). One blank microsphere population and four microsphere populations labeled with increasing amounts of Alexa Fluor® 488 fluorochrome were analyzed by flow cytometry to generate a fluorescence intensity standard curve. The microspheres were acquired using the same instrument settings used for analysis of the cell samples. For each cell sample, 1×10^6 cells were stained with 1000 nM Alexa Fluor® 488-conjugated anti-human CD47 mAb (B6H12) and analyzed by flow cytometry using the same fluorescence acquisition settings as those used for the MESF calibration microspheres.

Flow cytometry gating strategy:

The gating and quantification strategy was performed sequentially as follows:

1. **Singlet selection:** Events were displayed as FSC-H versus FSC-A, and a gate was applied around the diagonal singlet population to exclude cell doublets and aggregates.
2. **Viable-cell selection:** FSC-A was plotted against 7-AAD fluorescence, and the 7-AAD-negative population was selected to exclude dead or membrane-compromised cells.

3. **Cell population selection:** FSC-A versus SSC-A was used to identify the cell population of interest and exclude cellular debris and non-cellular events. The appropriate population was identified according to the characteristic FSC/SSC profile of each cell type analyzed.
4. **CD47 fluorescence analysis:** Alexa Fluor® 488 fluorescence was assessed within the gated singlet, viable-cell population. Fluorescence intensity was displayed as a histogram, and the median fluorescence intensity (MFI) was determined using FlowJo software.
5. **MESF-based quantification:** The MFI obtained for each cell population was converted to Alexa Fluor® 488 molecules of soluble fluorochrome equivalent (MESF) using the calibration curve generated from the Quantum™ Alexa Fluor® 488 MESF microspheres. The resulting MESF values were used to estimate cell-surface CD47 expression for each cell population.

Representative flow cytometry gating plots illustrating the sequential selection of singlet, viable cells and the respective cell populations, together with the MESF calibration curve, are provided below.

Representative Flow Cytometry Gating Plots and the MESF Calibration Curve for Quantification of Human CD47 Protein Expression Level (A549 Tumor Cells)

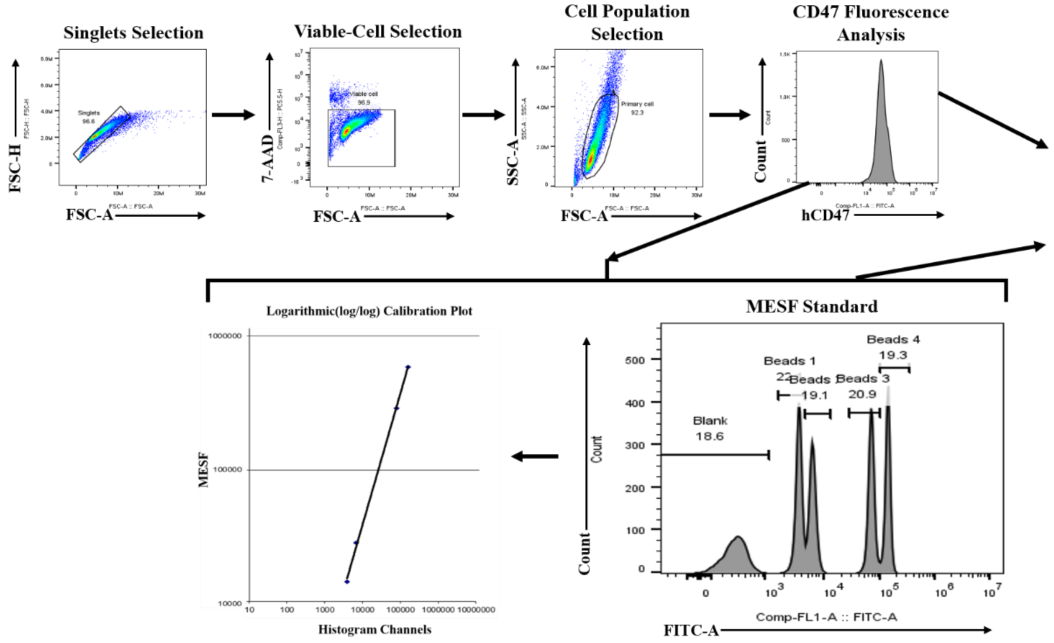

MESF-based quantification:

The MFI of each tumor cell samples compare with the bead standard curve. The measured fluorescence intensity was then converted to an absolute molecular count, providing an estimate of cell-surface CD47 protein expression.
